# The Oxidative Potential of Airborne Particulate Matter Research Trends, Challenges, and Future Perspectives—Insights from a Bibliometric Analysis and Scoping Review

**DOI:** 10.3390/antiox13060640

**Published:** 2024-05-24

**Authors:** Luis Felipe Sánchez, Loreto Villacura, Francisco Catalán, Richard Toro Araya, Manuel A. Leiva Guzman

**Affiliations:** Departamento de Química, Facultad de Ciencias, Universidad de Chile, Las Palmeras 3425, Ñuñoa, Santiago 7800003, Chile; luisfelipesanchezpuentes@gmail.com (L.F.S.); loreto.villacura@ug.uchile.cl (L.V.); francisco.catalan213@gmail.com (F.C.); rtoro81@uchile.cl (R.T.A.)

**Keywords:** airborne, particulate matter, oxidative potential, oxidative stress, paradigm

## Abstract

This study is a comprehensive analysis of the oxidative potential (OP) of particulate matter (PM) and its environmental and health impacts. The researchers conducted a bibliometric analysis and scoping review, screening 569 articles and selecting 368 for further analysis. The study found that OP is an emerging field of study, with a notable increase in the number of publications in the 2010s compared to the early 2000s. The research is primarily published in eight journals and is concentrated in a few academic and university-based institutions. The study identified key research hotspots for OP-PM, emphasizing the importance of capacity building, interdisciplinary collaboration, understanding emission sources and atmospheric processes, and the impacts of PM and its OP. The study highlighted the need to consider the effects of climate change on OP-PM and the regulatory framework for PM research. The findings of this study will contribute to a better understanding of PM and its consequences, including human exposure and its effects. It will also inform strategies for managing air quality and protecting public health. Overall, this study provides valuable insights into the field of OP-PM research and highlights the need for continued research and collaboration to address the environmental and health impacts of PM.

## 1. Introduction

Significant changes in the atmospheric composition have caused a dramatic paradigm shift in atmospheric chemistry and toxicological research [1,2]. These changes include not only an increase in the worldwide presence of potentially dangerous chemical pollutants, but also an increase in the accumulation of greenhouse gases [3]. These pollutants are harmful to human health and biodiversity [4,5,6]. The current age, dubbed the Anthropocene, is distinguished by large-scale human disruption of natural systems [7], and air pollution is a key concern that comes with this period [8,9].

Toxicologically, changes in atmospheric constituents have broad and profound implications [10,11]. Airborne particles, including fine and ultrafine particles and their constituents, as well as organic compounds, heavy metals, nitrogen and sulfur oxides, and ozone, among others, have potentially deleterious effects on human health [12,13]. These effects on health are subsequently manifested in an increase in morbidity and mortality rates [14,15,16]. Furthermore, many pollutants can accumulate in ecosystems and food chains, with potential wide-ranging adverse effects on both biodiversity and human health [17,18]. The World Health Organization (WHO) has highlighted air pollution as one of the most pressing global public health problems facing us today. In 2019, 99% of the global population was estimated to live in regions that do not meet WHO air quality guidelines, resulting in an estimated 6.7 million premature deaths per year [19].

During the past few decades, a growing body of epidemiologic evidence has established an incontrovertible link between the exposure to particulate matter (PM) and adverse health outcomes [20]. Therefore, the WHO has classified air pollution as a class 1 carcinogen, indicating a strong association, particularly between PM exposure and human carcinogenesis [21]. This correlation has led to a particular focus on studying PM, a complex mixture of solid and liquid particles that are suspended in the air, mainly due to its high impact on human health [22]. Robust correlations were identified between lung cancer progression and exposure to various PM components, including copper (Cu), zinc (Zn), sulfur (S), nickel (Ni), and potassium (K) [23]. Furthermore, increased mortality from various causes has been associated with exposure to compounds or elements such as nitrates, sulfates, and organic and elemental carbon [24,25]. Exposure to PM-bound elements, such as zinc (Zn), copper (Cu), and iron (Fe), has also been associated with chronic respiratory diseases, such as obstructive lung disease [26,27]. Furthermore, the incidence of preterm births and low birth weights may increase with exposure to PM, which contains polycyclic aromatic hydrocarbons (PAHs), organic carbon (OC), elemental carbon (EC), benzene, chromium (Cr), iron (Fe), nickel (Ni), sulfur (S), aluminum (Al), and titanium (Ti), from motor vehicle emissions and biomass burning [28,29,30]. However, it is important to recognize that efforts to directly link the chemical composition of PM with the underlying toxic mechanisms and health effects have had only partial success, in part because of the extraordinarily complex mixture of PM, which consists of hundreds or even thousands of compounds. This complexity makes it difficult, if not impossible, to fully identify and quantify PM, much less to determine and assess its health effects [31,32]. Furthermore, PM components can have synergistic and antagonistic interactions that cannot be adequately assessed from individual mass concentrations [33]. Recognizing these obstacles and limitations to the assessment of the effects of PM on human health, a novel approach has emerged. This approach is based on the fundamental premise that oxidative stress is a key driver in the physiological pathway that orchestrates both the health and disease outcomes that are induced by exposure to PM [34,35].

The harmful effects of PM toxicity are mainly attributed to the formation of free radicals, specifically reactive oxygen species (ROS) and reactive nitrogen species (RNS), induced by its components [36,37]. These radicals initiate a powerful chain reaction that can compromise the body’s antioxidant defenses, culminating in a state known as oxidative stress. Consequently, oxidative stress damages cellular macromolecules, potentially leading to apoptosis [38]. Two main mechanisms are thought to underlie PM exposure-induced oxidative stress: the first involves redox reactions that are triggered by redox-active PM constituents, including soluble transition metals, quinones, and polycyclic aromatic hydrocarbons (PAHs), which can deplete dissolved oxygen [35]; the second involves a biological reaction that is associated with cellular oxidative phosphorylation by sequential addition of electrons to dissolved oxygen—essentially, the biological generation of ROS in response to PM exposure [39]. Within this conceptual framework, the notion of oxidative potential (OP), or the redox activity of PM, emerges as a descriptor and metric of the relationship between the chemical composition of the PM, its physical properties, and the possible toxicological effects that it may exert on human health upon exposure [40]. Thus, OP is closely related to the biological mechanisms by which the adverse health effects of PM constituents can manifest themselves [41].

OP, a quantifiable measure of the ability of PM to oxidize a target molecule, is intrinsically linked to the generation of free radicals and the resulting oxidative stress, key aspects that can be evaluated using a variety of OP assays, both cellular and acellular [42,43]. There has been a noticeable shift toward the use of acellular assays for the quantification of OPs because of their relative simplicity in terms of analytical chemistry procedures, which is a contrast to those employing biological systems. This makes them advantageous for early, efficient, and direct environmental monitoring, potentially allowing for an earlier assessment of PM’s impact on human health [44]. The most widely used acellular methods for OP measurement include antioxidant depletion (such as ascorbic acid, uric acid, and glutathione), dichlorofluorescein, dithiothreitol, salicylic acid/benzoates, and electron paramagnetic resonance spectroscopy [42]. Some studies have shown statistically significant correlations between various OP assays and intracellular ROS production in in vitro and in vivo experiments, as well as with health outcomes in epidemiological studies. These observations suggest that OP-PM may serve as a reliable marker for PM that has a potential biological impact. Nevertheless, the elucidation of the most biologically relevant mechanisms that are responsible for ROS generation is still a critical challenge [45,46,47].

Considerable progress has been made in the measurement of OP in recent years. This progress is an important step toward establishing OP as an innovative and promising measure. Remarkable progress has been made in the design and standardization of toxicity assays, as methodologies have been refined [48,49]. As a result, our ability to quantify and interpret the effects of air pollutants has been greatly enhanced by the increased precision and reliability of studies focusing on the evaluation of OP, which has also provided the basis for conducting short- and long-term epidemiological assessments to characterize the toxicity of PM and its constituents [50,51]. Significant advances are being made in understanding how various components of particulate matter, including transition metals and organic compounds, can contribute to their oxidative potential and, hence, their ability to cause oxidative damage in human tissues [52]. These discoveries, along with advances in the standardization of toxicity assays, provide a solid framework for future research in this rapidly evolving field. Finally, we must emphasize that this progress also allows for the development of effective strategies to mitigate the harmful effects of air pollution on the health of the population.

The main objective of the present research is to identify the main points of interest in the study of OP-PM. This will be achieved through a careful bibliometric analysis of our current knowledge. As demonstrated in the academic literature, such bibliometric studies facilitate a clear delineation of scientific research trajectories and meticulously examine the current state of a given discipline or research area [53]. The chosen methodology, supported by an extensive bibliographic resource anthology, will facilitate the formation of a holistic view that combines both qualitative and quantitative aspects. The assessment includes aspects such as scientific productivity by country, corresponding authors’ countries, international collaboration, an analysis of core journals and journal distribution, most locally and globally cited documents, and the characteristics of institutional contribution. It also deciphers the current state of the art while identifying potential avenues for future research.

## 2. Materials and Methods

In this study, we utilized the 2018 extension of the PRISMA-ScR extension for Scoping Reviews (Preferred Reporting Items for Systematic reviews and Meta-Analyses extension for Scoping Reviews) framework. This framework comprises a series of well-established steps, incorporating clear inclusion and exclusion criteria, along with a rigorous quality assessment [54]. All of these steps were carefully followed in our work [55] (see Figure S1 for more details in Supplementary Materials). To ensure this, we adhered to a detailed PRISMA-ScR checklist. This checklist covers the most important elements of information that is needed to carry out a thorough and transparent review of the scope (see the Supplementary Material, BibOPPM_Checklist-ScR.pdf for details). Protocols for the scoping review, which provide a thorough discussion of each phase, are included in the Supplementary Material (BibOPPM_Protocol-ScR.pdf). The methodological aspects of this study are comprehensively documented in the Open Science Framework (OSF) registry, accessible at https://doi.org/10.17605/OSF.IO/SA6WB, accessed on 21 January 2024. The repository includes all documentation, as described in the previous paragraph. This is in accordance with the principles of quality, transparency, and reproducibility of this study. Brief descriptions of each of these steps are given below.

Step 1: *Definition of the field of study.* The focus of the research is the OP-PM, which determines the search strategy and database to be used.

Step 2: *Database Selection and Search Strategy*. The database selected in the present study is the Web of Science (WoS) Core Collection. This database, which is often used in such studies, integrates several databases, including the Science Citation Index Expanded (SCI-Expanded), the Social Sciences Citation Index (SSCI), the Arts & Humanities Citation Index (AHCI), and the Emerging Sources Citation Index (ESCI). The search strategy within the WoS was implemented through a series of seven meticulous search queries, each using unique groups of keywords that were agreed upon by consensus by all authors (see Table 1).

Searches #1, #2, and #3 correspond to independent queries that, for each set of keywords separately, include the fields tags title (TI), abstract (AB), and author keywords (AKs), linked by the Boolean operator “OR”. These searches are performed across all available issues and publication years (1975–present). In search #4, searches #1 and #2 are merged by using the Boolean operator “OR”. Next, in query #5, queries #4 and #5 are combined using the “AND” Boolean. Finally, in queries #6 and #7, the results are filtered by adding “articles” only and excluding articles published after 2022, respectively. At the end of the process, a database report was obtained.

Step 3: *Refinement of the search and/or eligibility criteria.* Duplicate verification using an automated tool and evaluation by three reviewers (LFS, FC, and LV) is used to refine the report database. These reviewers independently determine the eligibility of a report by examining the title, abstract, keywords of the author, and/or the full report. To be considered eligible for a report, the three reviewers must agree that the report is eligible. In case of disagreement, the report is given a “conflict” status and must be reviewed by a fourth reviewer (MALG) and discussed in a panel. The final decision on eligibility is made by a majority of reviewers. In this process, only reports that are directly related to OP-PM were included, that is, reports that involve measurements of OP in PM samples or evaluate and/or develop methods for measuring OP. Finally, reports where reviews were not within the scope of the search were excluded.

At the same time, during the review process, a questionnaire was used to gather additional information, with which homogenized keywords were assigned to each report (details of these keywords are provided in the Supplementary Material, Table S1). These keywords consider aspects such as author affiliation, study characteristics, study objectives, type of OP assay, complementary assessment method to OP, pollutants or variables other than OP, spatiotemporal scales, spatial scale, emission source contributing to OP, and potential implications of the study. To minimize the risk of bias and increase confidence in the results, homogenized keywords were discussed and agreed in a panel by all reviewers. Categorical variables were designed to represent a range of values and extract numerical data, such as spatiotemporal scales. Furthermore, an “other” option was included next to a text box in each question of the questionnaire to allow for comments regarding information not initially considered. All reviewers on the panel reviewed these comments. If the level of occurrence warranted it, new homogenized keywords were assigned, and the corresponding correction was applied to the reports that were reviewed previously. Comments with weighted occurrences of less than 1% were classified under the “other” option. This method enables the acquisition of more precise information, thus minimizing potential heterogeneity in the results of individual reports.

The data charting process was facilitated by a web-enabled collaborative review application, SWIFT ActiveScreener version 1.061.0791, provided by SCIOME [56]. This application allows for a double assessment of each report and a review by a panel of experts. Additionally, it includes a deduplication function that verifies the presence of duplicates when uploading to the report database based on the title, journal, and/or year of publication.

**Figure 1:**
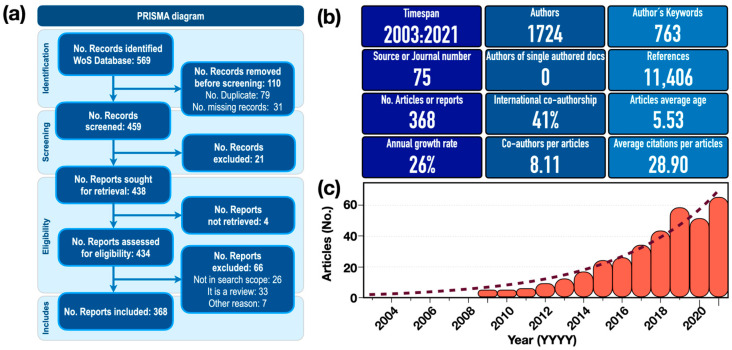
(**a**) The PRISMA-ScR flow chart used to search for articles in the WoS database. (**b**) Bibliometric summary metrics of the article collection. (**c**) Trends of articles published on the oxidative potential of airborne particulate matter.

Step 4: *Quantitative and qualitative analysis*. This analysis takes into account aspects related to authors, coauthors, journal sources, keywords, author affiliations, and countries or regions of authorship. The findings are systematized with a PRISMA flow diagram [57,58,59] (see Figure 1a).

VOSviewer version 1.6.19 [60] and the R-based web interface Bibliometrix version 3.2.1 [61] were used to construct and visualize bibliometric networks for quantitative and qualitative bibliometric analyses. Furthermore, MS Excel Mac version 16.59 [62] was used to tabulate and process the data. Within the R environment [63], the Ggplot 2 package R [64] was used to generate specific plots. This comprehensive approach integrates different tools and methods to elucidate patterns and networks and provides insightful representations of bibliographic data. When bibliographic data were incomplete or unclear, the missing fields (such as DOI, Author keywords, and Journals) were completed by extracting information directly from the available articles. Implementing this control measure improves the level of certainty and confidence in the information contained in the database.

Step 5: *Analysis of results*. Finally, Step 5 consists of analyzing and discussing the results. These are presented in the next section.

## 3. Results and Discussion

### 3.1. Descriptive Analysis of the Data

An initial collection of 569 articles was identified during the review of the research on OP-PM using the PRISMA-ScR approach. Subsequently, a sample of 368 articles (hereafter referred to as the “article collection”) was obtained, as shown in the flowchart (Figure 1a), with the 368 final articles obtained by exporting to an Excel bibliometrics file using Biblioshiny (see OSF registry at https://doi.org/10.17605/OSF.IO/SA6WB). Descriptive statistics can be used to carry out an initial exploration of the results. Figure 1b shows the main results of the article collection to be analyzed. A total of 1724 authors contributed to the article collection, with an average coauthorship rate per document of 8.11 and a coauthorship rate involving authors from different countries of approximately 41%. This reflects a strong degree of international collaboration on the subject. The average age of the article collection is 5.53 years, with an average citation rate of 28.93. These results demonstrate, first, the recent development of this field of research and, second, the great interest in the topic. Publications in the 2010s represent 66% of the total number of publications. This represents an increase of more than 24 times compared to the first decade of the 2000s. The number of publications showed an exponential growth trend of 26% per year (R^2^ = 0.95, see Figure 1c). The first article in the collection was published in 2003 (see database BibOP-PM-Bibliometrix.xlsx in OSF registry).

### 3.2. Descriptive Analysis of the Data

**Figure 2:**
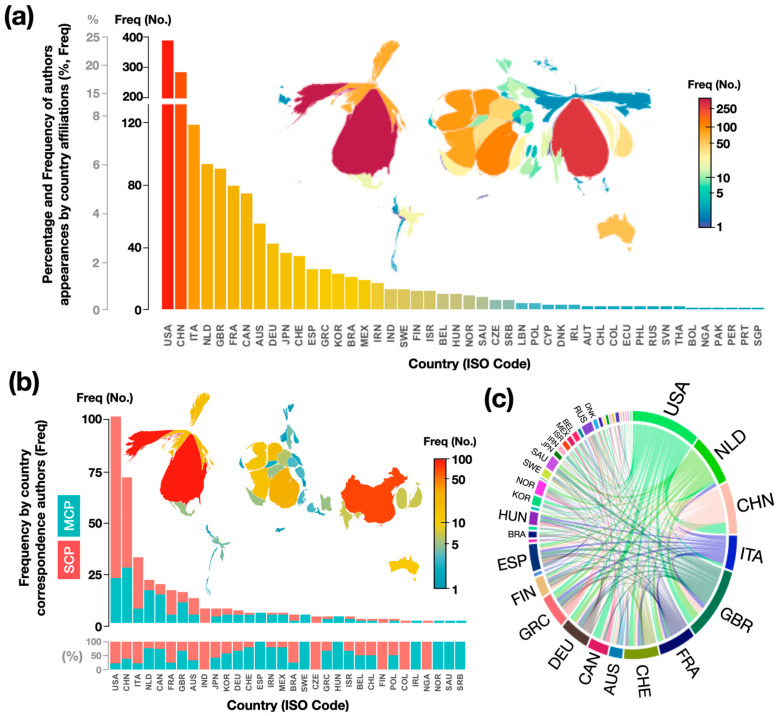
(**a**) Histogram and cartogram of geographical distribution of articles according to country of authors and coauthors, (**b**) histogram and cartogram of geographical distribution of articles according to country of corresponding author(s), and (**c**) origin–destination diagram showing collaboration between corresponding authors from one country with respect to others. Names of countries in ISO 3166-1 alpha-3 codes [65].

Figure 2a presents a cartogram-type map showing the geographical distribution of articles based on the country of affiliation of the authors and coauthors, using ISO 3166-1 alpha-3 country codes [65]. The size of each country is distorted based on the frequency of author appearances by country affiliation. Additionally, a histogram of the total number of authors who are affiliated with an institution in a given country is included in Figure 2a. Assuming a similar contribution of authors, the cartogram-type map shows a projection of each country in proportion to the number of authors and coauthors who are affiliated with that country. It is important to consider that not all authors contribute equally to the generation of each article. This difficulty arises from the fact that the information that is obtained from databases may not contain this information, or it may not be available in the articles. The results show the participation of authors who are affiliated with institutions from 46 countries. These countries are located on five continents. However, there are countries with zero productivity, including almost all of Africa (with the exception of NGA) and countries in the Middle East, Central Asia, and Southeast Asia. More than 62% are contributed by the top five countries. The authors from the USA contributed the most (25%). This was followed by CHN (18%), ITA (8%), NLD (6%), and GBR (6%). Figure 2b also shows the cartogram and histogram of the most productive countries according to the country of origin of the corresponding authors and the percentage of publications from multiple countries (MCP) and publications from a single country (SCP). The size of each country in the visualization is distorted by the frequency of corresponding authors. These results show that the most prominent countries according to the participation of authors from one country (Figure 2a) and according to the corresponding author are basically the same. This indicates that the countries with the highest production also had the highest number of articles published as countries of the corresponding authors. The five countries with the highest number of corresponding authors represent 66% of the contribution and correspond to the USA (27%), followed by CHN (19%), ITA (9%), NLD (6%), and CAN (5%); the first four coincide with the four most productive countries. NLD and CAN are the countries with the highest proportions of multicountry publications (MCP: 76% and 74%, respectively), indicating strong international collaboration. The lowest proportions are found in the USA and ITA (MCP: approximately 22%), suggesting that authors from these countries collaborate less with researchers from other countries. Figure 2c on origin–destination, which shows the collaboration of corresponding authors from one country with respect to other countries, shows that the sources of the articles are concentrated in North America (USA and CAN), Europe (NLD, ITA, GBR, FRA, and CHE), and East Asia (CHN).

### 3.3. Analysis of Core Journals and Journal Distribution

**Figure 3:**
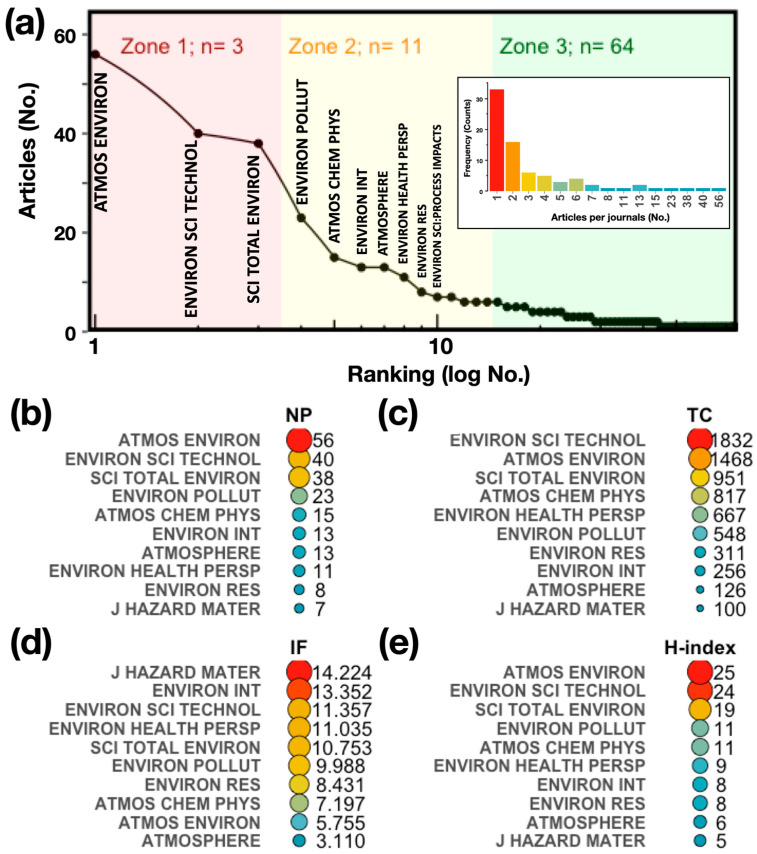
(**a**) Bradford’s law of most prominent sources. (**b**) Number of published articles (NP) per journal. (**c**) Total number of local citations (TCs) per journal. (**d**) Impact factor (IF) of the journal. (**e**) H-index of journal considering the articles from the collection on OP-PM.

The frequency of articles per journal shows how attractive it is for researchers to publish their research results in certain journals. The histogram of the distribution of articles published by journals is shown in Figure 3a, embedded figure. The results show that a total of 75 different journals have published articles in the field. Most of the publications were concentrated in a few sources. Overall, 45 journals published one to two papers, and only 8 journals published more than ten papers.

On the other hand, Bradford’s law was used to identify core sources [66]. This law describes a quantitative relationship between the journals and the articles that are contained in a collection on a given subject and states that the production of articles in journals is established by a highly uneven distribution, where most of the articles are concentrated in a small population of journals, while a small proportion of articles are spread over a large number of journals, following a distribution that is proportional to a ratio of 1 (zone 1):n (zone 2):n^2^ (zone 3). Here, zone 1 is the number of core journals that are preferred by researchers and therefore are most specific to a domain of knowledge. The results obtained by applying Bradford’s law, Figure 3a, to the article collection show that the “core zone” is composed of three sources with a total of 134 articles published, corresponding to 36% of the total number of articles; the second and third zones are composed of 11 and 64 sources, respectively.

The top 10 journals preferred by the authors were ranked according to the number of articles published (NP) in each journal (Figure 3b), followed by the total local citations (TCs) of the journals (Figure 3c), the impact factor (IF, Figure 3d), and the H-index (Figure 3e) that are associated with the article collection. This group of journals includes, as expected, the core group that predicts Bradford’s law (Figure 3a). The *ATMOS ENVIRON* journal stands out with a total of 15% of published articles (NP: 56), making it the journal chosen by the majority of authors in this field. It is followed by *ENVIRON SCI TECHNOL* (NP: 40), *SCI TOTAL ENVIRON* (NP: 38), and *ENVIRON POLLUT* (NP: 23). In terms of NP, the top 10 journals present an average of 22 published articles (CI95%: 12 to 32) and an average of 14, with their contribution making up almost 60% of the articles in the collection (Figure 3b). The TCs of this group of journals also stands out, with an average of 707 citations (CI95%: 348 to 1066, Figure 3b). It should be noted that an important aspect in the choice of the journal to be published is its IF. The top 10 journals have an average IF of 9 (CI95%: 7 to 11) and a mean of 10 (Figure 3d). Regarding the H-index, the top 10 journals have an average of 13 (CI95%: 8 to 17) and a mean of 10 (Figure 3e). The journal with the highest H-index is *ATMOS ENVIRON* (H-index: 25). The indexing categorization of the top 10 journals is mainly in the fields of “Environmental Sciences” and “Meteorology and Atmospheric Sciences”, and all of them are in the top five categories. In general, given their scope, the journals have published a large number of articles on atmospheric pollution, which, together with their high citation counts and IF and H indices, makes them attractive to researchers in this field and makes them the reference sources in this area of research.

### 3.4. Most Relevant Authors and Coauthorship Network

**Figure 4:**
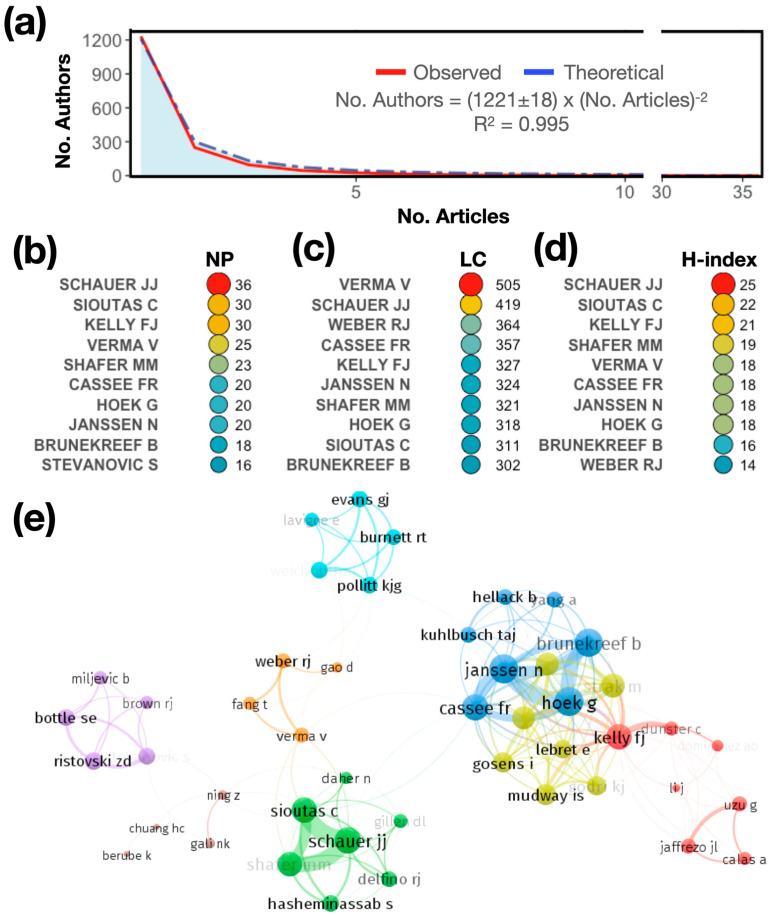
(**a**) Frequency distribution of publications using Lotka’s law. (**b**) Ranking of the most relevant authors according to the number of published articles (NP), (**c**) number of total local citations (LCs), and (**d**) H-index. (**e**) The cooperation networks of the articles produced by the authors.

In this section, we present the authors who have contributed the most to the knowledge in the field. First, the productivity of the authors is assessed using Lotka’s law of scientific productivity in Figure 4a. Lotka’s law considers that, regardless of the field that is analyzed, most of the production is concentrated in a limited number of authors [67] and is described as the No. Authors = C × (No. Articles)^−2^, where C is an arbitrary constant [68]. In the present study, the data adjusted for Lotka’s inverse square law show a good fit (R^2^ = 0.9995). In our case, production is diversified, with many authors having only one publication (1243 out of 1724), representing 72% of the total authors. The top 10 authors represent 1% of the total number of authors. This implies that they contribute at least 16 articles on the topic.

The 10 top authors by productivity are shown in Figure 4b–d. They are presented in descending order according to the number of published articles (NP, Figure 4b) and the number of local citations (LCs, Figure 4c), which measures how often an author who is included in this collection has been cited by articles that are also included in the collection. Finally, the H-index is presented (Figure 4d). The results show that, considering the NP, Schauer JJ is the most prolific, with a total of 36 published articles, followed by Sioutas C (NP: 30) and Kelly FJ (NP: 30). However, the predominance of authors is not uniform across the different indicators. Considering the publication impact in terms of citations (LCs), Verma V is the most important author, with 505 citations, followed by Schauer JJ (LC: 419), Weber RJ (LC: 364), and Cassee FR (LC: 357). In terms of the H-index, Schauer JJ is the top author (H-index: 25), followed by Sioutas C (H-index: 22) and Kelly FJ (H-index: 21).

Figure 4e shows the coauthorship network among the authors who have published on the topic studied. In coauthorship networks, the authors of the network are connected as nodes by coauthored scientific publications. The colors represent the working groups as clusters. Authors coauthoring more than one paper are grouped in the same cluster. In this case, there are clusters of four to seven authors, with a high level of coauthorship. For example, Verma V, Fang T, Gao D, and Weber RJ have a strong collaborative relationship, as shown by the orange cluster. Verma V seems to be the central author of this community of researchers. The topology of the collaborative network is as follows: There are groups with a high level of collaboration (blue, yellow, green, and red clusters), and others that are more dispersed. This is an indication that there is not much collaborative activity among the other clusters or working groups, so there is a need for an increase in collaborative activity to advance research in this area.

### 3.5. Characteristics of the Institutions’ Contribution

**Figure 5:**
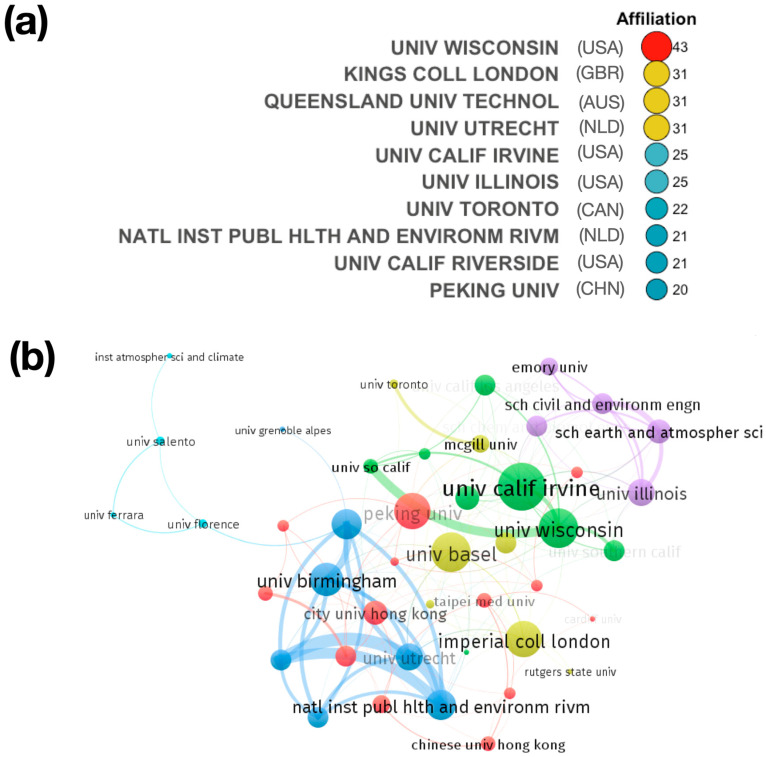
(**a**) Ranking of the most relevant research institutions in the area of OP-MP. (**b**) Cooperation networks for production by institutions.

The analysis of the distribution of institutions can aid in understanding the installed capacity for research in this field. In total, 532 institutions are associated with the authors in the collection. One or more authors are affiliated with an academic institution in 100% of the articles in the collection. However, 15% of the articles in the collection have at least one author affiliated with governmental organizations. In the article collections, there are virtually no authors from private institutions (2%) (see Figure S2 in Supplementary Materials). Figure 5a shows the 10 most productive institutions in terms of the number of publications. Approximately 18% of the publications were contributed by these top 10 institutions. They are mainly located in the USA, with four institutions, NLD with two institutions, followed by GBR, AUS, CAN, and CHN, with 1 institution each. UNIV WISCONSIN leads the ranking. It is followed by KINGS COLL LONDON and QUEENSLAND UNIV TECHNOL. The results clearly show the strong contribution of academic institutions (universities) in this area of research. There is no contribution from private sector institutions. The affiliation of the most productive authors is closely linked to these institutions. Finally, a stronger collaboration between institutions in the United States and between the United States and institutions in European countries can be seen in the collaboration network of the 50 most productive institutions (Figure 5b).

### 3.6. Most Locally and Globally Cited Documents

Table 2 shows the 10 most highly cited publications at the local level (LC), together with the number of global citations (GCs). In this case, the LCs take into account the number of times each publication was cited by other publications within the collection of articles, while the GCs measure the number of times each of the publications was cited in the entire WoS database; thus, it could take into account the relevance of a publication to other fields of study. The article by Charrier, JG, from 2012 turned out to be the most significant both locally (highest LCs) and globally for other fields (highest GCs) [48]. Charrier JG and Fang T stand out on the list, with two of their articles in the top 10 of the most relevant research in the field. In general, these papers are of interest, as they deal with aspects of OP methodologies and the effects of PM components in the different assays. On the other hand, the LCs/GCs ratio indicates that these articles not only have a local impact on the research topic but are also of interest in other research areas. This could be explained by the fact that OP is a metric that directly relates to the potential impact of PM on human health. Therefore, it is not surprising that these articles are of interest, for example, in the fields of toxicology and environmental epidemiology.

### 3.7. Research Hotspot Analysis

**Figure 6:**
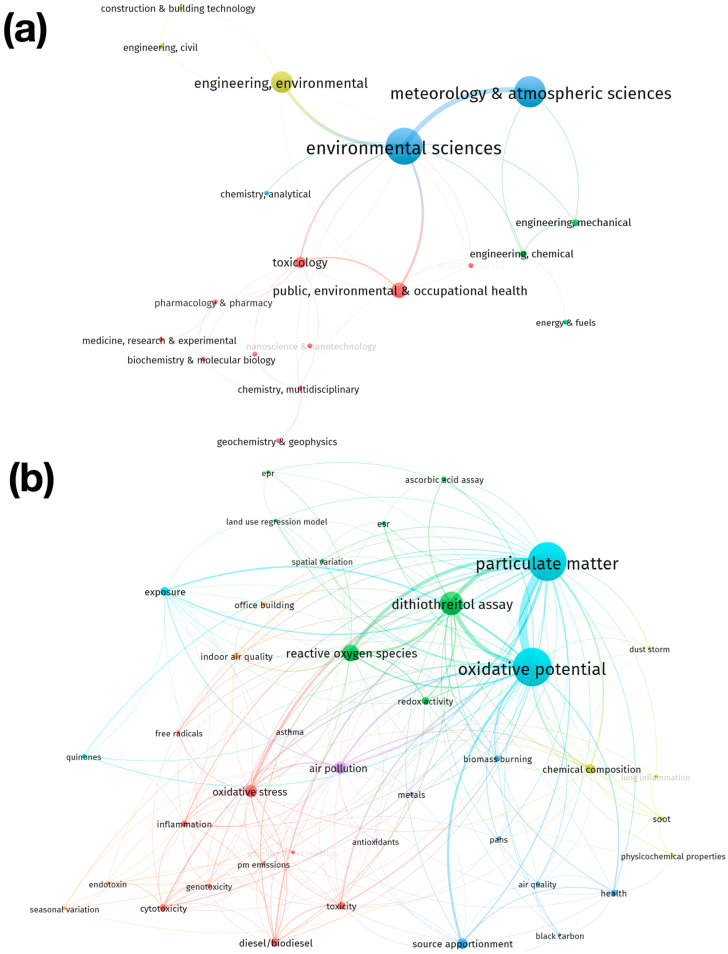
Analysis of hotspots in OP-PM from 2003 to 2021. (**a**) Co-occurrence network of WoS subject categories. (**b**) Co-occurrence network of author keywords.

Two types of analysis were performed for the global evaluation of OP-PM research hotspots. The first was based on the co-occurrence method between the thematic WoS categories (Figure 6a) and author keywords (Figure 6b) of the article collection with at least three interactions between them, grouped by the leading eigenvalue algorithm. The second analysis consisted of a detailed examination of each article in the collection, using a questionnaire to obtain and classify the information that is explicitly mentioned by the authors in relation to the main characteristics of the scope of the study, the objectives, the methods used, and the potential impact of the results obtained. The results are expressed in terms of the percentage of occurrence of each response. These percentages are weighted by the total number of articles in the collection. Both analyses allow us to understand the main characteristics of the research that has been conducted worldwide in relation to OP-PM and to identify possible research questions that have not yet been answered.

The results of analyzing the WoS subject categories indicate that the five main subject categories in which the articles in the collection were published correspond to the following (Figure 6a): “Environmental Sciences”, “Meteorology & Atmospheric Sciences”, “Engineering, Environmental”, “Public, Environmental & Occupational Health”, and “Toxicology”. The categories have been grouped into four clusters. These clusters can be characterized by the following topics: Cluster 1—Biological, chemical and health sciences (red cluster in Figure 6a): this is the group with the largest number of items and includes mainly categories that are related to chemical and biological health sciences, such as “biochemistry & molecular biology”, “chemistry, multidisciplinary”, “nanoscience & nanotechnology”, “chemistry, medical”, pharmacology and pharmacy, “toxicology”, and “public, environmental & occupational health”; Cluster 2—Engineering (green cluster in Figure 6a): this group includes categories such as “engineering, chemical”, “engineering, mechanical”, and “energy and fuel”; Cluster 3—Environmental science and chemical analysis (blue cluster in Figure 6a): includes categories such as “environmental science“, “meteorology and atmospheric science“, and “chemistry, analytical”. Finally, Cluster 4—Sustainable infrastructure (yellow color cluster in Figure 6a) includes categories such as “construction & building technology”, “engineering, civil”, and “engineering, environmental”.

However, the keyword analysis conducted by the authors shows that the five main keywords in the collection are particulate matter, oxidative potential, dithiothreitol assay, reactive oxygen species, and air pollution (Figure 6b). Keywords have been grouped into seven clusters. These clusters can be characterized as follows: Cluster 1: Fuels and toxicological health effects (red color cluster in Figure 6b): this group represents the cluster with the highest number of elements and includes keywords related to the emission of pollutants from the use of fuels such as “PM emission”, “gasoline direct injection”, or “diesel/biodiesel” and health effects such as “cytotoxicity”, “genotoxicity”, “toxicity”, “inflammation”, “free radicals”, and “oxidative stress”; Cluster 2—Oxidative potential assays and spatial variability (green color cluster in Figure 6b), which is the second largest group and includes keywords that are mainly related to assays for measuring oxidative potential such as “ascorbic acid assay”, “dithiothreitol assay”, “esr” (electron spin resonance), or “epr” (electron paramagnetic resonance) and spatial distribution variation models such as the “land use regression model” or “spatial variation”; Cluster 3—Biomass burning and its impacts (blue color cluster in Figure 6b), which includes keywords that are mainly related to biomass burning and its emissions such as “biomass burning”, “source apportionment”, “black carbon”, and “PAH” (polycyclic aromatic hydrocarbons) and its impacts such as “air quality” and “health”; Cluster 4—The physical characterization of particulate matter (yellow color cluster in Figure 6b), which includes keywords that are related to the characterization of particulate matter, such as “chemical composition” and “physicochemical properties”; Cluster 5—Air pollution and respiratory health (purple color cluster in Figure 6b): this group includes keywords such as “air pollution” and “asthma”, but on the other hand, it includes “antioxidants” and “metals”, which are keywords that can be related through the mechanism of oxidative stress; Cluster 6—Oxidative potential due to the exposure to particulate matter (cyan color cluster in Figure 6b), including keywords such as “exposure”, “particulate matter”, and “oxidative potential”. Finally, Cluster 7—Indoor pollution (orange color cluster in Figure 6b) includes keywords that are mainly related to indoor or workplace pollution, such as “indoor air quality” and “office building”.

The main findings from the application of the questionnaire to each of the articles collected are described below, as are the results of our analysis. Figures supporting this analysis and a brief description of the categories used are provided in the supplementary materials (see Tables S1–S12 and Figures S2–S14 in Supplementary Materials and/or BibOPPM-Result-ScR.xlsx for result database of the scoping review):

**Main characteristics of the scope of the study** (see Table S3 and Figure S3 in the Supplementary Materials). The primary emphasis of the research that is encapsulated in the article collection centers on evaluating the OP by utilizing environmental PM samples, which constitute a significant 71% of the entire dataset, referred to here as “Ambient”. Succeeding this, artificially created samples under regulated conditions, labeled “laboratory-generated samples”, account for a substantial 28% of the research. Lastly, the analysis includes samples that are procured from enclosed residential or occupational settings, denoted “Indoor”, and this forms 10% of the overall investigation. Together, these three aspects provide the structural foundations for field research. Recognizing the different proportions of these data sources is important, as it underscores the distinct importance of each source in this field. The focus of the study on examining the OP in real-world situations is highlighted by the heavy reliance on environmental samples. In addition, laboratory and indoor study samples provide valuable controlled data and contextual information, respectively.

**Objectives of the study** (see Table S4 and Figure S4 in the Supplementary Materials). Regarding the objectives of the studies, the first and most common is to “analyze the causes of air pollution and/or oxidizing potential (OP)” (71%). This involves the evaluation and characterization of zones where air pollution, air quality, or OP is affected or driven by comparable parameters (e.g., emission sources, dispersion conditions, deposition, or atmospheric chemistry that explain a spatial or temporal variability of OP in a specific area) or emission sources. The delimitation of areas where air pollution/air quality or OP is influenced/triggered by similar parameters or emission sources (e.g., sources with similar temporal variations, meteorological factors) is also an aim. The second most common focus is on “exposure assessment” (38%), which often involves determining the amount, duration, and frequency of human exposure by employing both mass-based exposure estimates and various OP metrics. Finally, a significant number of studies focus on the “evaluation of OP measurement methods” (26%). This could involve comparing the reliability and accuracy of different measurement techniques or establishing new methodologies to improve the accuracy of OP measurement.

**Methods used, OP assays, and chemical characterization** (see Tables S5–S8 and Figures S5–S8 in the Supplementary Materials). In terms of the type of OP assay used, acellular assays have emerged as the predominant method used by researchers in article collections, accounting for 86% of all tests and significantly outweighing the use of cellular assays, which accounts for only 21%. The five most commonly used OP assays, listed in descending order of prevalence, are the dithiothreitol assay (DTT: 57%), the ascorbic acid assay (AA: 25%), the dichlorofluorescein assay (DFCH: 21%), the reduced glutathione assay (GSH: 14%), and the electron paramagnetic/spin resonance assay (EPR/ESR: 11%). This preference for acellular assays is not surprising given their distinct advantages over cellular assays. They are more efficient and cost-effective, because they provide rapid results, require less stringent environmental conditions, and lend themselves to automation. However, despite the current dominance of acellular assays, research is beginning to shift toward the use of in vivo models. This transition is primarily driven by an increased understanding of the biological mechanisms that are related to PM. By authentically replicating complex biological interactions, in vivo models provide a more holistic and accurate assessment of the effects of PM.

All studies quantify the concentration of various air pollutants, such as PM or others (with 100% “concentration”), and a significant proportion evaluate their chemical composition (85%) as a complementary assessment method to OP. Toxicity is of tertiary concern, with a total toxicity of 39%. In general, these studies are aimed at correlating OP with chemical or physical parameters of the concentration of PM and/or gases, meteorological variables, analysis of emission sources, and others. However, of the large number of pollutants or variables that have been prominently featured in OP studies, five stand out due to their relative prevalence. These include PM, which tops the list with an overwhelming 90% representation. Next, in descending order, are “Elemental Composition” with 61% and “Carbon Compounds” with a substantial frequency of 51%. Further down the list, “Anions and Cations” appear, with 30%. Finally, the “Biological Endpoint” completes our list, with 25%. Each of these elements show a significant presence within this environmental context. This underscores their relevance in the overall analytical framework and in understanding the drivers of OP.

**PM samples’ geographical origin and spatiotemporal scales of the studies** (see Tables S9–S11 and Figures S9–S13 in Supplementary Materials). According to the results described in Section 3.2, the three main continents are Europe, with 35% of the samples, Asia, with 33%, and North America, with 32% of the samples, regarding the geographical origin of the PM samples. Among them, the United States, China, and Italy stand out, representing 25%, 21%, and 11% of the total sample, respectively. This suggests that there is a growing interest in this area of research, especially in developed countries compared to other geographical areas. In addition, an analysis of the emission sources that contribute to the oxidative potential (OP) emerges, and five major contributors emerge. First and foremost, “traffic” emerges as the most significant, accounting for 61% of OP. This is followed by “domestic heating—wood burning”, with 26%. This shows the influence of domestic activities on the OP. The category “industrial and commercial emissions” accounts for 22%. This underlines the role of the industrial emission source in the OP. Interestingly, “unspecified biomass burning” accounts for 21% of the OP. This suggests a significant influence of uncharacterized biomass burning. Finally, indicating the significant role of the environment itself in PM emissions and impacts, “natural sources” contribute 19%.

Regarding the spatiotemporal variation in the studies, we categorize the temporal scales from the most extended to the least. We find that 14% of the studies cover “More than a year–1 year”, 33% cover “Year—Season”, 16% are conducted over “3 months–1 month”, and 11% range from “three weeks to a few hours”. For the spatial scale, ordered from the most extensive to the least extensive, we find that 1% of the studies are conducted at a “global” scale, 5% at a “regional” scale, a substantial 60% at a “local” scale, and 35% at a “micro” scale. By systematically categorizing temporal and spatial scales, this observation provides a clear picture of how studies are distributed across different spatiotemporal scales.

The geographic origin and spatiotemporal expansion of PM sample studies is important in understanding the relationship between composition, temporal variability, and oxidative potential (OP), which varies significantly from region to region. These scales also suggest that the impact and implications of OP can differ markedly depending on both the time frame and the spatial context, reinforcing the need for multidimensional research approaches. Therefore, insightful information on pollutant sources can be obtained by understanding the geographical origin and providing the ability to assess the long- and short-term effects of the source and composition of PM in the OP.

**Potential impact of the study** (see Table S12 and Figure S14 in Supplementary Materials): The study primarily reveals valuable, application-oriented perspectives in the health sector, a component which is referred to here as “human health”, which accounts for an estimated 75% of the studies’ impacts. These results underscore a substantial correlation of OP with health outcomes on the usefulness of the exposure metric over mass concentration. Subsequently, the regulatory and legal sphere, referred to as “regulatory and legal”, gains substantial impact, comprising approximately 25% of the potential ramifications. This facet relates to the usefulness of OP as a surrogate measure for PM exposure in the formulation and implementation of mitigation strategies for the reduction in PM pollution. It also promotes the establishment of air quality standards that are more health-based, as opposed to being based solely on physicochemical parameters such as mass concentration. Finally, we address the elucidation of the intrinsic mechanisms that are associated with the OP metric, an area referred to as “understanding the OP mechanism”, which accounts for approximately 15% of the research implications. These insights are related to understanding the driving factors of OP in relation to the chemical–physical characteristics of PM, including emission sources, chemical composition, and size, as well as meteorological and orographic variables related to the dynamics and transport of pollutants.

The results of these studies deeply emphasize the potential implications of OP in a variety of sectors. The breadth of the scope of OPs and their implications in these areas imply a complex interweaving of diverse disciplines. This underscores the essential role of interdisciplinary collaboration and understanding in the pursuit of a deeper understanding and application of the results of OP research in the real world.

### 3.8. Future Research Directions and Remarks

The field of atmospheric science, particularly the study of particulate matter (PM) and its oxidative potential (OP), is critical for understanding its effects on human health and the environment. PM has been recognized as a trigger for the generation of cellular reactive oxygen species (ROS), which results in oxidative stress and a wide range of adverse health consequences. In comparison to the mass concentration of PM, the OP of PM has been accepted as a more biologically relevant parameter for assessing human exposure. Nonetheless, there are still significant unanswered issues and current debates in this field, demanding further research.

There is a pressing need for capacity enhancement and innovation in the measurement capacities of OP in terms of future research directions. This includes the development of precise, faster measurement methodologies as well as the development of cutting-edge instrument platforms for real-time monitoring. Capacity-building efforts should focus on the spread of experience and knowledge in order to strengthen air pollution control methods and improve air quality management practices.

Effective air quality management requires a thorough understanding of the effects of emission sources on PM and OP. Future research should concentrate on determining the sources of the oxidative stress that is caused by ambient PM and analyzing how weather influences emissions and atmospheric processes. This involves a comprehensive chemical and physical characterization of PM emitted from various sources, as well as the advancement of statistical tools for data interpretation.

Personal exposure to varied surroundings and activities is critical in determining the health effects of OP-PM. The focus of such research should be on quantifying the geographical and temporal changes of atmospheric elements that affect OP, as well as evaluating personal exposure to OP-PM in a variety of circumstances. This needs epidemiologic and toxicological investigations, as well as the development of precise measurement tools.

The study of new contaminants, particularly nanoparticles, is an important path for future research. To uncover the physicochemical properties and toxicity of nanoparticles and design effective measures to reduce their influence on human health and ecosystems, specialized research is necessary.

Furthermore, the interaction between climate change and its impacts on OP-PM requires careful consideration. It is critical to understand how extreme weather events affect OP-PM and to analyze the potential health and environmental consequences under various climatic conditions. Such research should try to measure emissions and clarify atmospheric processes, providing a framework for future mitigation efforts.

Combating air pollution demands a multifaceted strategy that integrates societal, legal, and policy frameworks. Policy makers can develop holistic plans to combat PM pollution by combining knowledge from atmospheric science and the social sciences, with an emphasis on equitable reduction and protecting vulnerable groups.

An interdisciplinary research strategy is required for a thorough knowledge of the environmental and health effects of PM and OP. Comprehensive investigations addressing the many facets of the source–exposure–dose–response paradigm for OP are required. Insights from various disciplines, such as chemistry, biology, public health, socioeconomic studies, and legal frameworks, will be used to considerably improve future research on OP-PM and inspire strong mitigation solutions. Further investigation is necessary to understand the correlation between OP-PM and biological endpoints in order to understand the underlying biological mechanisms of the health-related effects of PM.

In a broader sense, the present study emphasizes the need for OP-PM research in understanding the effects of PM on human health and the ecosystem. It highlights the importance of interdisciplinary collaboration and the integration of research across the exposure–risk assessment–risk management continuum, with an ultimate goal of protecting public health and the environment. The OP metric emerges as a more trustworthy proxy for evaluating PM exposure, requiring its incorporation into air quality management strategies.

### 3.9. Study Limitations: Strengths and Weaknesses

A bibliometric analysis is a powerful research tool that quantitatively evaluates the scientific literature in a specific field. It can map research hotspots, track the evolution of the field, and identify key trends, influential authors, institutions, and countries. Additionally, it can highlight the most cited articles, indicating studies that have had a significant impact. However, there are limitations to bibliometric analysis that can lead to biased interpretations or erroneous conclusions. These limitations arise from the completeness of the databases used, as they may exclude relevant studies that are published in other databases. Furthermore, databases evolve over time, which can result in variations in the articles that are included or excluded. The methodology used to collect articles also plays a crucial role, as a search strategy based on specific keywords and Boolean strings may inadvertently exclude or include relevant articles. Inconsistencies between the keywords and the actual content of the articles can occur, biasing the results. 

The strength of this study lies in its methodology, which ensures the reliability and validity of the conclusions. A panel evaluated the articles, and specialized software tools were used for analysis. The selection criteria for the search and the visualization of results were carefully considered throughout the research. The study followed rigorous processes, redundant steps, and verification to obtain accurate results. 

## 4. Conclusions

The present study provides a comprehensive analysis of the current research on OP-PM (the oxidative potential of particulate matter). Through a bibliometric analysis and scoping review approach, this study aims to offer a strategic framework for the advancement of the field and address perspectives on emerging trends.

The analysis of the 368 selected articles on OP-PM reveals robust international collaboration, with an average coauthorship rate of 8.11 per document and a cross-country coauthorship rate of approximately 41%. The average age of the article collection is 5.53 years, indicating the recent evolution of the field. The citation rate is significantly elevated at 28.93, indicating a substantial interest in the topic. The number of publications in the 2010s increased 24 times compared to the early 2000s, representing an exponential annual growth rate of 26%. This highlights the growing importance of PM-OP research.

The study identifies a small group of 8 journals that represent more than ten published articles, while the remaining content is spread over 75 different sources. The top ten journals account for approximately 60% of the total collection. Environmental Sciences and Meteorology and Atmospheric Sciences are the main disciplines of these journals. The study also identifies the top 10 most productive authors. The analysis of the coauthor network reveals different levels of collaboration among the authors, highlighting the importance of increased collaboration for further progress.

The study finds that 18% of the publications come from the top 10 institutions, indicating a concentration of research in a limited number of institutions, mainly academic institutions and universities. The study also highlights the global dimension of OP-PM research, with significant collaboration between institutions in the United States and European countries. The research has relevance to broader disciplines such as toxicology and environmental epidemiology, indicating its importance beyond local boundaries.

The key global research hotspots on OP-PM are identified, including environmental sciences; meteorology and atmospheric sciences; environmental engineering; public, environmental and occupational health; and toxicology. The research is grouped into four key clusters: “biological, chemical and health sciences”; “engineering”; “environmental science and chemical analysis”; and “sustainable infrastructure”. Future research directions are suggested, including strengthening capacity building and innovation, developing advanced OP measurement instrumentation, and fostering interdisciplinary collaboration. This study emphasizes the correlation between OP and health outcomes, supporting OP as a potential metric for the development of more health-centric air quality standards and PM mitigation strategies.

Overall, the study highlights the growing importance of OP-PM research and the need for increased collaboration and interdisciplinary approaches. It identifies key contributors, institutions, and journals in the field and provides insights into research hotspots and future directions. This study contributes to a more comprehensive understanding of the risks that are associated with PM and supports the development of targeted mitigation strategies to protect public health and environmental sustainability.

## Figures and Tables

**Table 1:** Keyword sets and search strategy used in the study (see for details table footnotes).

**Keyword Set #1**	
(“pollut*” AND air) OR (atmosp* AND pollut*) OR (“bad air quality”); (“Ambient air pollution”) OR (“quali*” NEAR air)	
**Keyword Set #2**	
(“partic* matter” OR “fine particulate” OR particulate OR “ultrafine partic*” OR “ultrafine partic*” OR “partic* pollut*” OR aeroso* OR Size segreg* OR Ozone OR Smog OR (“photochemical” NEAR smog) OR “nitrogen *oxide*” OR “sulfur *oxide*” OR “carbon monoxide” OR “heavy metal*” OR “volatile organic gases” OR “organic gases” OR dioxi* OR fura* OR “polycyclic aromatic hydrocarbon*” OR “Polychlorinated biphenyl*” OR “persistent organic pollutant*” OR “volatile organic compound*” OR “quinone*” OR AA*depletion OR GSH*depletion OR “Congo Red” OR “DCF” OR “DCFH-DA” OR “2-deoxyribose” OR DHE OR DTT OR ESR OR EPR OR Luminol OR CRAT OR “2′,7′-dichlorofluorescin” OR “dithiothreitol” OR “acid ascorbic” OR “Dihydroethidium” OR “glutathione” OR “electron spin resonance” OR “electron paramagnetic resonance”)	
**Keyword Set #3**	
(oxidative NEAR/5 potential)	
**Search** **No.**	**Search query**
1	TI = (Keyword Set #1) OR AB = (Keyword Set #1) OR AK = (Keyword Set #1) with the options Search in: Web of Science Core Collection; Edition: All; Publication date: All years (1975–present).
2	TI = (Keyword Set #2) OR AB = (Keyword Set #2) OR AK = (Keyword Set #2) with the options Search in: Web of Science Core Collection; Edition: All; Publication date: All years (1975–present).
3	TI = (Keyword Set #3) OR AB = (Keyword Set #3) OR AK = (Keyword Set #3) with the options Search in: Web of Science Core Collection; Edition: All; Publication date: All years (1975–present).
4	#1 OR #2 (Merge Search N°1 and Search N°2)
5	#3 AND #4 (Combine Search N°3 and Search N°4)
6	[Refine] by Document Types: Article
7	[Exclude] by Publication Years: 2022–present

Table footnotes: Quotation marks (“”) are employed to mandate a search for an exact phrase; an asterisk (*) denotes any number of characters at the end of a word; NEAR/x serves as the proximity operator, wherein the /x component determines the maximum number of words permissible between two key terms; OR is utilized in searches to amalgamate multiple search terms. The expression enclosed within parentheses is prioritized for execution. TI (Title) conducts searches within the Title field of a record. AB (Abstract) searches within the Abstract field of a record. AK (Author Keywords) searches within the Author Keywords field of a record.

**Table 2:** Top 10 relevant publications in research on OP-PM based on the number of local citations (LCs). In each case, the number of global citations (GCs) and the percentage of the LCs/GCs ratio are indicated.

Ranking (No.)	Most Locally Cited Documents (Authors, Year [Reference])	Journal Abbreviation	Digital Object Identifier DOI	LC (No.)	GC (No.)	LC/GC (%)
1	Charrier & Anastasio, 2012 [48]	*Atmos. Chem. Phys.*	https://doi.org/10.5194/acp-12-9321-2012	137	273	50
2	Fang et al., 2016 [69]	*Atmos. Chem. Phys.*	https://doi.org/10.5194/acp-16-3865-2016	84	143	59
3	Verma et al., 2012 [70]	*Environ. Sci. Technol.*	https://doi.org/10.1021/es302484r	73	193	38
4	Yang et al., 2014 [41]	*Atmos. Environ.*	https://doi.org/10.1016/j.atmosenv.2013.10.049	62	107	58
5	Delfino et al., 2013 [34]	*J. Expo. Sci. Env. Epidemiol.*	https://doi.org/10.1038/jes.2013.25	60	130	46
6	Fang et al., 2015 [51]	*Atmos. Meas. Tech.*	https://doi.org/10.5194/amt-8-471-2015	57	98	58
7	Steenhof et al., 2011 [71]	*Part. Fibre Toxicol.*	https://doi.org/10.1186/1743-8977-8-26	52	215	24
8	Charrier et al., 2015 [72]	*Atmos. Chem. Phys.*	https://doi.org/10.5194/acp-15-2327-2015	48	67	72
9	Saffari et al., 2014 [73]	*J. Environ. Sci. Health Part A*	https://doi.org/10.1080/10934529.2014.854677	46	68	68
10	Biswas et al., 2009 [74]	*Environ. Sci. Technol.*	https://doi.org/10.1021/es9000592	41	127	32

## Data Availability

Data supporting the findings of this study are available online. (i) the Protocol for Scoping Review (stored under the filename ‘BibOPPM_Protocol-ScR.pdf’), (ii) the PRISMA-ScR Checklist, which is the Checklist of Preferred Reporting Items for Systematic Reviews and Meta-Analyses extension for Scoping Reviews (available as ‘BibOPPM_Checklist-ScR.pdf’), (iii) a database aggregating articles that meet the predefined eligibility criteria (denoted as ‘BibOP-PM-Bibliometrix.xlsx’), and (iv) the results of the scoping review database (documented in ‘BibOPPM_Results-ScR.pdf’) are available online under an Open Science Foundation (OSF) Generalized Systematic Review Registration Form at: https://doi.org/10.17605/OSF.IO/SA6WB.

## Supplementary Materials

The following supplementary materials will be made available: https://www.mdpi.com/article/10.3390/antiox13060640/s1, Supplementary Material on Scoping Review Finding. Tables and figures included in the Supplementary Material: Table S1. Set of homogenized keywords assigned to each article according to the specific characteristics of the study through panel review (further details, a brief description, and examples can be found in Tables S2–S12). Table S2. The homogenized keywords used to extract author affiliations from the article collection. Table S3. The homogenized keywords used to extract main characteristic of the scope of the study from the article collection, brief descriptions, and examples. Table S4. The homogenized keywords used to extract the objectives of the study from the article collection, brief descriptions, and examples. Table S5. The normalized keywords used related to type of OP assay from article collection, brief descriptions, and examples. Table S6. The normalized keywords used related to OP assay from article collection, target molecule or method name, measurement principle, and reference. Table S7. The normalized keywords used related to evaluation method complementary to the OP, brief descriptions, and examples. Table S8. The normalized keywords used related to contaminant or variable other than OP, brief descriptions, and examples. Table S9. The normalized keywords used related to emission source from article collection, brief descriptions, and examples. Table S10. The normalized keywords used related to temporal scales of the study, brief descriptions, and examples. Table S11. The normalized keywords used related to spatial scales of the study, brief descriptions, and examples. Table S12. The homogenized keywords used to extract potential impact from the study from article collection, brief descriptions, and examples. Figure S1. A visual summary of the article screening process using the PRIS-MA-ScR extension for Scoping Reviews (Preferred Reporting Items for Systematic reviews and Meta-Analyses extension for Scoping Reviews) flow diagram (source: author’s elaboration based on PRISMA). Figure S2. Histogram of occurrences related to author’s affiliation, weighted by total collected articles (see Table S2 for additional information). Figure S3. Histogram of occurrences related to main characteristic of the scope of the study, weighted by total collected articles (see Table S3 for additional information). Figure S4. Histogram of occurrences related to objectives of the study, weighted by total collected articles (see Table S4 for additional information). Figure S5. Histogram of occurrences related to type of OP assay, weighted by total collected articles (see Table S5 for additional information). Figure S6. Histogram of occurrences related to OP assay, weighted by total collected articles (see Table S6 for additional information). Figure S7. Histogram of occurrences related to evaluation method complementary to the OP, weighted by total collected articles (see Table S7 for additional information). Figure S8. Histogram of occurrences related to contaminant or variable other than OP, weighted by total collected articles (see Table S8 for additional information). Figure S9. Histogram of occurrences related to continent of PM samples, weighted by total collected articles. Figure S10. Histogram of occurrences related to country of PM samples, weighted by total collected articles. Figure S11. Histogram of occurrences related to emission source, weighted by total collected articles (see Table S9 for additional information). Figure S12. Histogram of occurrences related to temporal scales, weighted by total collected articles (see Table S10 for additional information). Figure S13. Histogram of occurrences related to spatial scales of the study, weighted by total collected articles (see Table S11 for additional information). Figure S14. Histogram of occurrences related to potential impacts from the study, weighted by total collected articles (see Table S12 for additional information).

## References

[1] Crutzen P.J., Lelieveld J. (2001). Human Impacts on Atmospheric Chemisty. Annu. Rev. Earth Planet. Sci..

[2] NASEM (2016). The Future of Atmospheric Chemistry Research: Remembering Yesterday, Understanding Today, Anticipating Tomorrow.

[3] Crutzen P.J., Ehlers E., Krafft T. (2006). The “Anthropocene”. Earth System Science in the Anthropocene.

[4] Alan Pounds J., Bustamante M.R., Coloma L.A., Consuegra J.A., Fogden M.P.L., Foster P.N., La Marca E., Masters K.L., Merino-Viteri A., Puschendorf R. (2006). Widespread Amphibian Extinctions from Epidemic Disease Driven by Global Warming. Nature.

[5] Islam N., Dihingia A., Khare P., Saikia B.K. (2020). Atmospheric Particulate Matters in an Indian Urban Area: Health Implications from Potentially Hazardous Elements, Cytotoxicity, and Genotoxicity Studies. J. Hazard. Mater..

[6] Li Y., Muñoz-Ibañéz F., Maldonado-Alcaíno A., Jack D., Yan B., Xu L., Acuña M., Leiva-Guzman M., Valdés A., Cáceres D.D. (2022). Cancer Burden Disease Attributable to PM2.5 and Health Risk by PM2.5-Bound Toxic Species in Two Urban Chilean Municipalities. Aerosol Air Qual. Res..

[7] Lewis S.L., Maslin M.A. (2015). Defining the Anthropocene. Nature.

[8] Chen B., Kan H. (2008). Air Pollution and Population Health: A Global Challenge. Environ. Health Prev. Med..

[9] Lelieveld J. (2017). Clean Air in the Anthropocene. Faraday Discuss..

[10] Englert N. (2004). Fine Particles and Human Health—A Review of Epidemiological Studies. Toxicol. Lett..

[11] Offer S., Hartner E., Di Bucchianico S., Bisig C., Bauer S., Pantzke J., Zimmermann E.J., Cao X., Binder S., Kuhn E. (2022). Effect of Atmospheric Aging on Soot Particle Toxicity in Lung Cell Models at the Air–Liquid Interface: Differential Toxicological Impacts of Biogenic and Anthropogenic Secondary Organic Aerosols (SOAs). Environ. Health Perspect..

[12] Belleudi V., Faustini A., Stafoggia M., Cattani G., Marconi A., Perucci C.A., Forastiere F. (2010). Impact of Fine and Ultrafine Particles on Emergency Hospital Admissions for Cardiac and Respiratory Diseases. Epidemiology.

[13] Yang Y., Ruan Z., Wang X., Yang Y., Mason T.G., Lin H., Tian L. (2019). Short-Term and Long-Term Exposures to Fine Particulate Matter Constituents and Health: A Systematic Review and Meta-Analysis. Environ. Pollut..

[14] Díaz-Robles L.A., Fu J.S., Vergara-Fernández A., Etcharren P., Schiappacasse L.N., Reed G.D., Silva M.P. (2014). Health Risks Caused by Short Term Exposure to Ultrafine Particles Generated by Residential Wood Combustion: A Case Study of Temuco, Chile. Environ. Int..

[15] Jerrett M., Burnett R.T., Beckerman B.S., Turner M.C., Krewski D., Thurston G., Martin R.V., van Donkelaar A., Hughes E., Shi Y. (2013). Spatial Analysis of Air Pollution and Mortality in California. Am. J. Respir. Crit. Care Med..

[16] Sommar J.N., Hvidtfeldt U.A., Geels C., Frohn L.M., Brandt J., Christensen J.H., Raaschou-Nielsen O., Forsberg B. (2021). Long-Term Residential Exposure to Particulate Matter and Its Components, Nitrogen Dioxide and Ozone—A Northern Sweden Cohort Study on Mortality. Int. J. Environ. Res. Public Health.

[17] Ali H., Khan E., Ilahi I. (2019). Environmental Chemistry and Ecotoxicology of Hazardous Heavy Metals: Environmental Persistence, Toxicity, and Bioaccumulation. J. Chem..

[18] Greaver T.L., Sullivan T.J., Herrick J.D., Barber M.C., Baron J.S., Cosby B.J., Deerhake M.E., Dennis R.L., Dubois J.-J.B., Goodale C.L. (2012). Ecological Effects of Nitrogen and Sulfur Air Pollution in the US: What Do We Know?. Front. Ecol. Environ..

[19] World Health Organization Ambient (Outdoor) Air Pollution [Fact Sheet]. https://www.who.int/news-room/fact-sheets/detail/ambient-(outdoor)-air-quality-and-health.

[20] Brook R.D., Rajagopalan S., Pope C.A., Brook J.R., Bhatnagar A., Diez-Roux A.V., Holguin F., Hong Y., Luepker R.V., Mittleman M.A. (2010). Particulate Matter Air Pollution and Cardiovascular Disease. Circulation.

[21] IARC Working Group on the Evaluation of Carcinogenic Risks to Humans (2015). Outdoor Air Pollution.

[22] Morakinyo O., Mokgobu M., Mukhola M., Hunter R. (2016). Health Outcomes of Exposure to Biological and Chemical Components of Inhalable and Respirable Particulate Matter. Int. J. Environ. Res. Public Health.

[23] Raaschou-Nielsen O., Beelen R., Wang M., Hoek G., Andersen Z.J., Hoffmann B., Stafoggia M., Samoli E., Weinmayr G., Dimakopoulou K. (2016). Particulate Matter Air Pollution Components and Risk for Lung Cancer. Environ. Int..

[24] Cakmak S., Dales R.E., Angelica Rubio M., Blanco Vidal C. (2011). The Risk of Dying on Days of Higher Air Pollution among the Socially Disadvantaged Elderly. Environ. Res..

[25] Wang Y., Shi Z., Shen F., Sun J., Huang L., Zhang H., Chen C., Li T., Hu J. (2019). Associations of Daily Mortality with Short-Term Exposure to PM2.5 and Its Constituents in Shanghai, China. Chemosphere.

[26] Gehring U., Beelen R., Eeftens M., Hoek G., de Hoogh K., de Jongste J.C., Keuken M., Koppelman G.H., Meliefste K., Oldenwening M. (2015). Particulate Matter Composition and Respiratory Health: The PIAMA Birth Cohort Study. Epidemiology.

[27] Zhang Z., Weichenthal S., Kwong J.C., Burnett R.T., Hatzopoulou M., Jerrett M., van Donkelaar A., Bai L., Martin R.V., Copes R. (2021). A Population-Based Cohort Study of Respiratory Disease and Long-Term Exposure to Iron and Copper in Fine Particulate Air Pollution and Their Combined Impact on Reactive Oxygen Species Generation in Human Lungs. Environ. Sci. Technol..

[28] Balidemaj F., Flanagan E., Malmqvist E., Rittner R., Källén K., Åström D.O., Oudin A. (2022). Prenatal Exposure to Locally Emitted Air Pollutants Is Associated with Birth Weight: An Administrative Cohort Study from Southern Sweden. Toxics.

[29] Pedersen M., Gehring U., Beelen R., Wang M., Giorgis-Allemand L., Andersen A.-M.N., Basagaña X., Bernard C., Cirach M., Forastiere F. (2016). Elemental Constituents of Particulate Matter and Newborn’s Size in Eight European Cohorts. Environ. Health Perspect..

[30] Wilhelm M., Ghosh J.K., Su J., Cockburn M., Jerrett M., Ritz B. (2011). Traffic-Related Air Toxics and Preterm Birth: A Population-Based Case-Control Study in Los Angeles County, California. Environ. Health.

[31] Kelly F.J., Fussell J.C. (2012). Size, Source and Chemical Composition as Determinants of Toxicity Attributable to Ambient Particulate Matter. Atmos. Environ..

[32] Li T., Yu Y., Sun Z., Duan J. (2022). A Comprehensive Understanding of Ambient Particulate Matter and Its Components on the Adverse Health Effects Based from Epidemiological and Laboratory Evidence. Part. Fibre Toxicol..

[33] Kastury F., Smith E., Juhasz A.L. (2017). A Critical Review of Approaches and Limitations of Inhalation Bioavailability and Bioaccessibility of Metal(Loid)s from Ambient Particulate Matter or Dust. Sci. Total Environ..

[34] Delfino R.J., Staimer N., Tjoa T., Gillen D.L., Schauer J.J., Shafer M.M. (2013). Airway Inflammation and Oxidative Potential of Air Pollutant Particles in a Pediatric Asthma Panel. J. Expo. Sci. Environ. Epidemiol..

[35] Ghio A.J., Carraway M.S., Madden M.C. (2012). Composition of Air Pollution Particles and Oxidative Stress in Cells, Tissues, and Living Systems. J. Toxicol. Environ. Health.

[36] Tuet W.Y., Fok S., Verma V., Tagle Rodriguez M.S., Grosberg A., Champion J.A., Ng N.L. (2016). Dose-Dependent Intracellular Reactive Oxygen and Nitrogen Species (ROS/RNS) Production from Particulate Matter Exposure: Comparison to Oxidative Potential and Chemical Composition. Atmos. Environ..

[37] Molina C., Toro A. R., Manzano C., Canepari S., Massimi L., Leiva-Guzmán M. (2020). Airborne Aerosols and Human Health: Leapfrogging from Mass Concentration to Oxidative Potential. Atmosphere.

[38] Montiel-Dávalos A., de Jesús Ibarra-Sánchez M., Ventura-Gallegos J.L., Alfaro-Moreno E., López-Marure R. (2010). Oxidative Stress and Apoptosis Are Induced in Human Endothelial Cells Exposed to Urban Particulate Matter. Toxicol. Vitr..

[39] Soberanes S., Urich D., Baker C.M., Burgess Z., Chiarella S.E., Bell E.L., Ghio A.J., De Vizcaya-Ruiz A., Liu J., Ridge K.M. (2009). Mitochondrial Complex III-Generated Oxidants Activate ASK1 and JNK to Induce Alveolar Epithelial Cell Death Following Exposure to Particulate Matter Air Pollution. J. Biol. Chem..

[40] Molina C., Manzano C.A., Toro A. R., Leiva G M.A. (2023). The Oxidative Potential of Airborne Particulate Matter in Two Urban Areas of Chile: More than Meets the Eye. Environ. Int..

[41] Yang A., Jedynska A., Hellack B., Kooter I., Hoek G., Brunekreef B., Kuhlbusch T.A.J., Cassee F.R., Janssen N.A.H. (2014). Measurement of the Oxidative Potential of PM2.5 and Its Constituents: The Effect of Extraction Solvent and Filter Type. Atmos. Environ..

[42] Bates J.T., Fang T., Verma V., Zeng L., Weber R.J., Tolbert P.E., Abrams J.Y., Sarnat S.E., Klein M., Mulholland J.A. (2019). Review of Acellular Assays of Ambient Particulate Matter Oxidative Potential: Methods and Relationships with Composition, Sources, and Health Effects. Environ. Sci. Technol..

[43] Murphy M.P., Bayir H., Belousov V., Chang C.J., Davies K.J.A., Davies M.J., Dick T.P., Finkel T., Forman H.J., Janssen-Heininger Y. (2022). Guidelines for Measuring Reactive Oxygen Species and Oxidative Damage in Cells and in Vivo. Nat. Metab..

[44] Ayres J.G., Borm P., Cassee F.R., Castranova V., Donaldson K., Ghio A., Harrison R.M., Hider R., Kelly F., Kooter I.M. (2008). Evaluating the Toxicity of Airborne Particulate Matter and Nanoparticles by Measuring Oxidative Stress Potential—A Workshop Report and Consensus Statement. Inhal. Toxicol..

[45] Øvrevik J. (2019). Oxidative Potential Versus Biological Effects: A Review on the Relevance of Cell-Free/Abiotic Assays as Predictors of Toxicity from Airborne Particulate Matter. Int. J. Mol. Sci..

[46] Guascito M.R., Lionetto M.G., Mazzotta F., Conte M., Giordano M.E., Caricato R., De Bartolomeo A.R., Dinoi A., Cesari D., Merico E. (2023). Characterisation of the Correlations between Oxidative Potential and in Vitro Biological Effects of PM10 at Three Sites in the Central Mediterranean. J. Hazard. Mater..

[47] Lu Y., Su S., Jin W., Wang B., Li N., Shen H., Li W., Huang Y., Chen H., Zhang Y. (2014). Characteristics and Cellular Effects of Ambient Particulate Matter from Beijing. Environ. Pollut..

[48] Charrier J.G., Anastasio C. (2012). On Dithiothreitol (DTT) as a Measure of Oxidative Potential for Ambient Particles: Evidence for the Importance of Soluble Transition Metals. Atmos. Chem. Phys..

[49] Eiguren-Fernandez A., Kreisberg N., Hering S. (2017). An Online Monitor of the Oxidative Capacity of Aerosols (o-MOCA). Atmos. Meas. Tech..

[50] Bates J.T., Weber R.J., Abrams J., Verma V., Fang T., Klein M., Strickland M.J., Sarnat S.E., Chang H.H., Mulholland J.A. (2015). Reactive Oxygen Species Generation Linked to Sources of Atmospheric Particulate Matter and Cardiorespiratory Effects. Environ. Sci. Technol..

[51] Fang T., Verma V., Guo H., King L.E., Edgerton E.S., Weber R.J. (2015). A Semi-Automated System for Quantifying the Oxidative Potential of Ambient Particles in Aqueous Extracts Using the Dithiothreitol (DTT) Assay: Results from the Southeastern Center for Air Pollution and Epidemiology (SCAPE). Atmos. Meas. Tech..

[52] Gao D., Ripley S., Weichenthal S., Godri Pollitt K.J. (2020). Ambient Particulate Matter Oxidative Potential: Chemical Determinants, Associated Health Effects, and Strategies for Risk Management. Free Radic. Biol. Med..

[53] José de Oliveira O., Francisco da Silva F., Juliani F., César Ferreira Motta Barbosa L., Vieira Nunhes T., Kunosic S., Zerem E. (2019). Bibliometric Method for Mapping the State-of-the-Art and Identifying Research Gaps and Trends in Literature: An Essential Instrument to Support the Development of Scientific Projects. Scientometrics Recent Advances.

[54] Page M.J., Moher D., Bossuyt P.M., Boutron I., Hoffmann T.C., Mulrow C.D., Shamseer L., Tetzlaff J.M., Akl E.A., Brennan S.E. (2021). PRISMA 2020 Explanation and Elaboration: Updated Guidance and Exemplars for Reporting Systematic Reviews. BMJ.

[55] Mak S., Thomas A. (2022). Steps for Conducting a Scoping Review. J. Grad. Med. Educ..

[56] Howard B.E., Phillips J., Tandon A., Maharana A., Elmore R., Mav D., Sedykh A., Thayer K., Merrick B.A., Walker V. (2020). SWIFT-Active Screener: Accelerated Document Screening through Active Learning and Integrated Recall Estimation. Environ. Int..

[57] Boers M. (2018). Graphics and Statistics for Cardiology: Designing Effective Tables for Presentation and Publication. Heart.

[58] Mayo-Wilson E., Li T., Fusco N., Dickersin K. (2018). Practical Guidance for Using Multiple Data Sources in Systematic Reviews and Meta-Analyses (with Examples from the MUDS Study). Res. Synth. Methods.

[59] Stovold E., Beecher D., Foxlee R., Noel-Storr A. (2014). Study Flow Diagrams in Cochrane Systematic Review Updates: An Adapted PRISMA Flow Diagram. Syst. Rev..

[60] van Eck N.J., Waltman L. (2010). Software Survey: VOSviewer, a Computer Program for Bibliometric Mapping. Scientometrics.

[61] Aria M., Cuccurullo C. (2017). Bibliometrix: An R-Tool for Comprehensive Science Mapping Analysis. J. Informetr..

[62] (2022). MS-Excel.

[63] (2022). RStudio.

[64] Wickham H., Gentleman R., Hornik K., Parmigiani G. (2009). Ggplot2: Elegant Graphics for Data Analysis.

[65] International Organization for Standardization ISO 3166 Country Codes—Online Browsing Platform. https://www.iso.org/iso-3166-country-codes.html.

[66] Brookes B.C. (1985). “Sources of Information on Specific Subjects” by S.C. Bradford. J. Inf. Sci..

[67] López-Fernández M.C., Serrano-Bedia A.M., Pérez-Pérez M. (2016). Entrepreneurship and Family Firm Research: A Bibliometric Analysis of An Emerging Field. J. Small Bus. Manag..

[68] Chung K.H., Pak H.S., Cox R.A.K. (1992). Patterns of Research Output in the Accounting Literature: A Study of the Bibliometric Distributions. Abacus.

[69] Fang T., Verma V., Bates J.T., Abrams J., Klein M., Strickland M.J., Sarnat S.E., Chang H.H., Mulholland J.A., Tolbert P.E. (2016). Oxidative Potential of Ambient Water-Soluble PM_2.5_ in the Southeastern United States: Contrasts in Sources and Health Associations between Ascorbic Acid (AA) and Dithiothreitol (DTT) Assays. Atmos. Chem. Phys..

[70] Verma V., Rico-Martinez R., Kotra N., King L., Liu J., Snell T.W., Weber R.J. (2012). Contribution of Water-Soluble and Insoluble Components and Their Hydrophobic/Hydrophilic Subfractions to the Reactive Oxygen Species-Generating Potential of Fine Ambient Aerosols. Environ. Sci. Technol..

[71] Steenhof M., Gosens I., Strak M., Godri K.J., Hoek G., Cassee F.R., Mudway I.S., Kelly F.J., Harrison R.M., Lebret E. (2011). In Vitro Toxicity of Particulate Matter (PM) Collected at Different Sites in the Netherlands Is Associated with PM Composition, Size Fraction and Oxidative Potential—The RAPTES Project. Part. Fibre Toxicol..

[72] Charrier J.G., Richards-Henderson N.K., Bein K.J., McFall A.S., Wexler A.S., Anastasio C. (2015). Oxidant Production from Source-Oriented Particulate Matter—Part 1: Oxidative Potential Using the Dithiothreitol (DTT) Assay. Atmos. Chem. Phys..

[73] Saffari A., Daher N., Shafer M.M., Schauer J.J., Sioutas C. (2014). Seasonal and Spatial Variation in Dithiothreitol (DTT) Activity of Quasi-Ultrafine Particles in the Los Angeles Basin and Its Association with Chemical Species. J. Environ. Sci. Health.

[74] Biswas S., Verma V., Schauer J.J., Cassee F.R., Cho A.K., Sioutas C. (2009). Oxidative Potential of Semi-Volatile and Non Volatile Particulate Matter (PM) from Heavy-Duty Vehicles Retrofitted with Emission Control Technologies. Environ. Sci. Technol..

