# Supplementary material for: The Oxidative Potential of Airborne Particulate Matter Research Trends, Challenges, and Future Perspectives—Insights from a Bibliometric Analysis and Scoping Review
Source: Antioxidants (Basel). 2024 May 24;13(6):640. doi: 10.3390/antiox13060640 (PMC11200927; doi:10.3390/antiox13060640)
Supplement: Supplementary file 1 [file antioxidants-13-00640-s001.zip › antioxidants-2750235-supplementary.pdf]

antioxidants

Supplementary material

Oxidative Potential of Airborne Particulate Matter Research Trends, Challenges, and Future Perspectives - Insights from a Bibliometric Analysis and Scoping Review

Luis Felipe Sánchez, Loreto Villacura, Francisco Catalán, Richard Toro A., Manuel A. Leiva G. *

Departamento de Química, Facultad de Ciencias, Universidad de Chile, Las Palmeras 3425, Ñuñoa, Santiago, Chile.

***Correspondence: manleiva@uchile.cl**

Index of Tables

Table S1. Set of homogenized keywords assigned to each article according to the specific characteristics of the study through panel review (a more details, a brief description, and examples, in the Tables S2 to 12) 4

Table S2. The homogenized keywords used to extract author affiliations from the article collection 5

Table S3. The homogenized keywords used to extract extraction of main characteristic of the scope of the study from the article collection, brief descriptions, and examples 6

Table S4. The homogenized keywords used to extract extraction of the objectives of the study from the article collection, brief descriptions, and examples. 7

Table S5. The normalized keywords used related to type of OP assay from article collection, brief descriptions, and examples. 9

Table S6. The normalized keywords used related to OP assay from article collection, target molecule or method name, measurement principle, and reference. 10

Table S7. The normalized keywords used related to evaluation method complementary to the OP, brief descriptions, and examples. 12

Table S8. The normalized keywords used related to contaminant or variable other than OP, brief descriptions, and examples. 13

Table S9. The normalized keywords used related to emission source from article collection, brief descriptions, and examples. 16

Table S10. The normalized keywords used related to temporal scales of the study, brief descriptions, and examples. 17

Table S11. The normalized keywords used related to spatial scales of the study, brief descriptions, and examples. 18

Table S12. The homogenized keywords used to extract potential impact from the study from article collection, brief descriptions, and examples. 19

Index of Figures

Figure S1. A visual summary of the article screening process using the PRISMA (Preferred Reporting Items for Systematic Reviews and Meta-Analyses) flow diagram (Source: Author's elaboration based on PRISMA.). 3

Figure S2. Histogram of occurrences related to author's affiliation, weighted by total collected articles (see Table S2 for additional information). 5

Figure S3. Histogram of occurrences related to main characteristic of the scope of the study, weighted by total collected articles (see Table S3 for additional information). 6

Figure S4. Histogram of occurrences related to objectives of the study, weighted by total collected articles (see Table S4 for additional information). 8

Figure S5. Histogram of occurrences related to type of OP assay, weighted by total collected articles (see Table S5 for additional information). 9

Figure S6. Histogram of occurrences related to OP assay, weighted by total collected articles (see Table S6 for additional information). 11

Figure S7. Histogram of occurrences related to evaluation method complementary to the OP, weighted by total collected articles (see Table S7 for additional information) 12

Figure S8. Histogram of occurrences related to contaminant or variable other than OP, weighted by total collected articles (see Table S8 for additional information). 14

Figure S9. Histogram of occurrences related to continent of PM samples, weighted by total collected articles. 14

Figure S10. Histogram of occurrences related to country of PM samples, weighted by total collected articles. 15

Figure S11. Histogram of occurrences related to emission source, weighted by total collected articles (see Table S9 for additional information). 16

Figure S12. Histogram of occurrences related to temporal scales, weighted by total collected articles (see Table S10 for additional information). 17

Figure S13. Histogram of occurrences related to spatial scales of the study, weighted by total collected articles (see Table S11 for additional information). 18

Figure S14. Histogram of occurrences related to potential impacts from the study, weighted by total collected articles (see Table S12 for additional information). 19

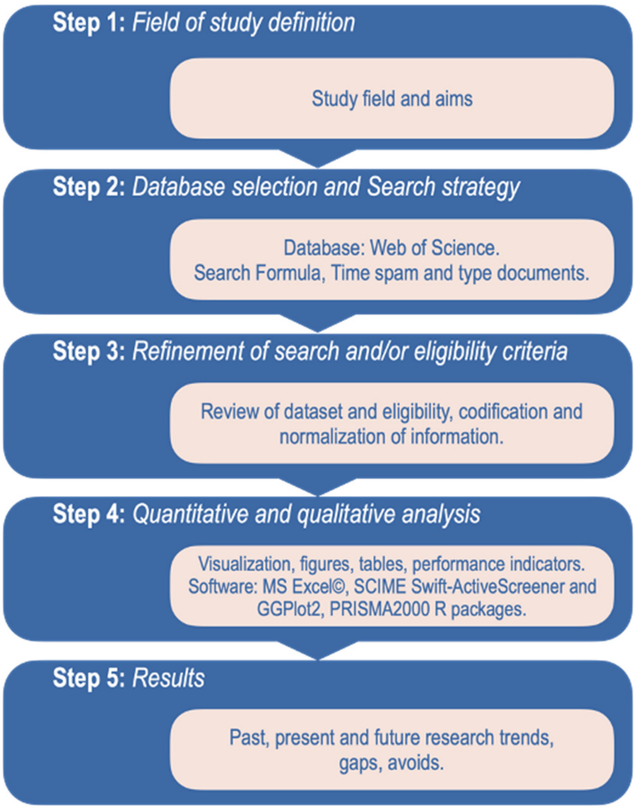

Figure S1. A visual summary of the article screening process using the PRISMA (Preferred Reporting Items for Systematic Reviews and Meta-Analyses) flow diagram (Source: Author's elaboration based on PRISMA).

Table S1. Set of homogenized keywords assigned to each article according to the specific characteristics of the study through panel review (a more details, a brief description, and examples, in the Tables S2 to 12).

Theme	Explanation	Homogenized keywords by theme
Authors' affiliation type.	Refers to the professional or institutional association of an author at the time of the paper's publication, typically indicating where the bulk of the research was conducted.	Academy (ACA), Public sector (PUB), Private sector (PRI), International organizations (INO), Other (OT01).
Main characteristic of the scope of the study.	Refers to the boundaries or parameters within which the study is conducted.	Ambient (AMB), Indoor (IND), Reference materials (RMA), laboratory generated samples (LGS), Commercial materials (COM), Other (OT02).
Objectives of the study.	Refer to the explicit statements outlining what the study aims to achieve; they define the specific goals or research questions that guide the investigation.	Spatial distribution (SPD); Exposure assessment (EXA); Analysis of causes of air pollution and/or OP (ACP); Modeling air pollution (MOA); Source apportionment (SAP); Evaluation of OP measurement methodologies (EOM); Management tool (MAT), Other (OT03).
Type of OP assay.	Refers to laboratory assay to assess the OP in cellular or acellular environments.	Cellular (CEL), Acellular (ACE).
OP assays.	Refers to analytical methodologies used to quantify the OP PM. These assays measure the capacity of PM to catalyze redox reactions, providing insight into the toxicity of airborne particulates.	Electron Paramagnetic/Spin Resonance (EPR/ESR), Dithiothreitol (DTT), Ascorbic acid (AA), Cytochrome C (CYC), Salicylate (SLC), 9,10-bis (phenyl ethynyl) anthracene-nitroxide (BPEA), Chemiluminescent Reductive Acridinium Triggering Assay (CRAT), Dichlorodihydrofluorescein diacetate (DFCH), Reduced glutathione (GSH), Dihydroethidium (DHE), Luminol (LML), Respiratory tract lining fluid assay (RTLF), Uric acid (UA), Plasmid scission assay (PSA), Markers of oxidative stress (MOS), Other (OT05).
Evaluation method complementary to the OP.	Refers to exploration and integration of supplementary analytic and modeling methodologies to enhance existing operational procedures in the studies.	Concentrations (CON), Chemical composition (CHE), Physical measurements of pollutants (PHM), Meteorological measurements (MET), Biological composition (BIO), Toxicological (TOX), Epidemiological (EPI), Computer Modeling (MOD), Satellite approach (SAT), Others unclassified (OT06).
Contaminant or variable other than OP.	Refers to any pollutant or quantity other than OP that may potentially affect the atmospheric composition or processes considered in the study.	Particulate matter (PM), Elemental composition (ECM), carbon compounds (CCP), Nitrogen oxides (NOX), Ozone (O3), Sulfur oxides (SOX), Carbon oxides (COX), Volatile/semivolatile organic compounds (VOC), Anions and cations (ANC), Biological measurement (BIO), Biological end point (BIE), Emergent contaminants (EMC), Polycyclic aromatic hydrocarbons (PAH), Secondary organic aerosols (SOA), Quinones (QUI), Other (OT07), No information (NA07).
Continent in which the study of PM samples.	Refers to the geographic categorization of research locations. This distinction allows for the exploration of regional variations in PM pollution characteristics.	North America, South America, Europe, Africa, Asia, Oceania, Antarctica.
Country in which the study of PM samples.	Refers to the country where particulate PM samples are collected for research purposes.	Country (ISO 3166-1 alpha-3 code).
Emission source that contributes to the OP.	Refers to the main emission source contributing to the OP PM, often anthropogenic. It is intrinsically linked to the chemical composition of PM and related to its redox activity.	Domestic heating/wood burning (DOM), Traffic (TRA), Industrial and commercial emissions (ICE), Mining (MIN), Power generation (POW), Agriculture (AGR), Natural sources (NAT), Wildfires (WIL), Non-exhaust emissions-traffic related (NOT), Unspecified biomass burning no specification (BBN), Others (OT10), No information (NA10).
Temporal scales.	Refers to the length of time or span of time over which the data are being observed in the study.	More than a year - 1 year (YRM), Year - Season (YRS), 3 months - 1 month (3M1), 3 weeks - Hours (3WH), Other (OT10), No information (NA).
Spatial scales.	Refers to a parameter that delineates the dimensions of spatial phenomena or processes and thus allows them to be measured and interpreted in an effective way.	Micro-scale (MIC), Local (LOC), Regional (REG), Global (GLO), No information (NA).
Potential impacts of the study.	Refers to the possible, multiple outcomes, changes or impacts that research may have across a range of disciplines or sectors.	Human health (HEA), Ecological (ECO), Climate (CLIM), Technological development (TEC), Economic (ECN), Social and Cultural (SOC), Regulatory and Legal (REG), Understanding OP mechanism (UOM), Other (OT04).

Table S2. The homogenized keywords used to extract author affiliations from the article collection.

**Affiliation**	**Brief descriptions**	**Examples**
Academy	Affiliation in academic institutions.	Universities, institutes, research centers, and other academic institutions.
Public sector	The affiliation corresponds to all the administrative organizations of the State.	State ministries, public institutions, government offices and other administrative organizations.
Private sector	Affiliation corresponds to a group of non-governmental organizations and/or individuals.	Companies, private laboratories, foundations, and other groups of non-governmental organizations and/or individuals.

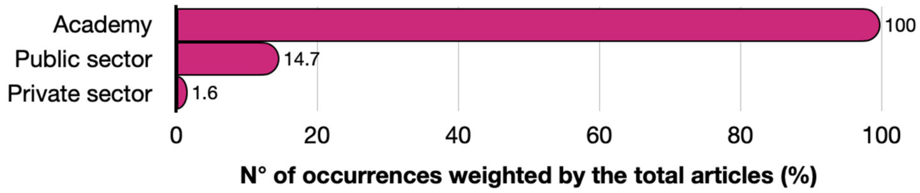

Figure S2. Histogram of occurrences related to author's affiliation, weighted by total collected articles (see Table S2 for additional information).

Table S3. The homogenized keywords used to extract extraction of main characteristic of the scope of the study from the article collection, brief descriptions, and examples.

**Main characteristic of the scope of study**	**Brief descriptions**	**Examples**
Ambient	Outdoor measurement of pollutants.	Study of long-range transport of pollutants; source apportionment; and assessment of air quality from outdoor sampling (urban or rural).
Indoor	Measurement of pollutants in indoor environments.	Assess the impact on indoor air quality from the use of fireplaces, stoves, or ovens; indoor/outdoor air exchange.
Laboratory generated samples	Artificially generated samples in controlled environments.	Study of emissions from exhaust pipes or pellet combustion; evaluation of engine performance; study of the mechanism of particle formation in reaction chambers.
Reference materials	Use materials or substances with properly characterized and certified chemical composition and properties.	Use of standard reference materials developed by the National Institute Standards and Technology (NIST) to certify or validate a measurement.
Commercial materials	Use of materials or substances purchased from a commercial supplier that do not necessarily meet the quality standards of a reference material.	Study the effect of using different filter materials on OP assays.
Other	Other study characteristics that have not yet been a considered and has a low percentage of occurrence.	Study characteristics not previously considered.

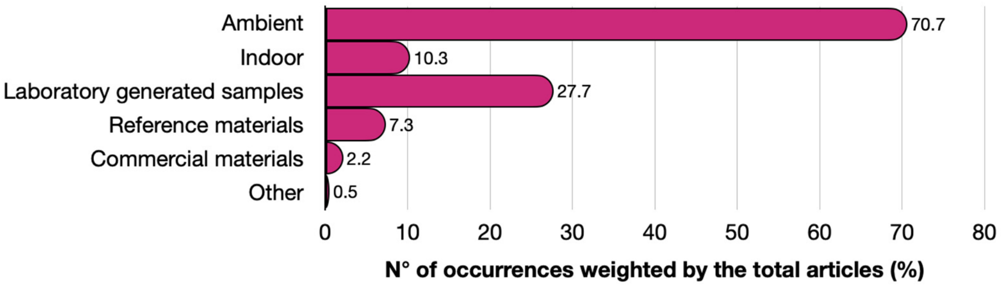

Figure S3. Histogram of occurrences related to main characteristic of the scope of the study, weighted by total collected articles (see Table S3 for additional information).

Table S4. The homogenized keywords used to extract extraction of the objectives of the study from the article collection, brief descriptions, and examples.

Objectives of the study	Brief descriptions	Examples
Analysis of causes of air pollution and/or OP	Delimitation of areas where air pollution/air quality or OP is influenced/triggered by similar parameters or emission sources (e.g., sources with similar temporal variations, meteorological factors).	Study of emission sources, dispersion conditions, deposition, or atmospheric chemistry that explains a pollution event or OP in a specific area.
Exposure assessment	Estimate pollutant effects on human health, ecosystems, and specific plant species.	Study of how OP-related air pollution affects human respiratory health, biodiversity; and the growth/performance of certain vegetal species in urban environments.
Evaluation of OP measurement methodologies	Comparison of results from different OP assays; testing of the effect of extraction methods; and design and analytical validation of OP measurement methods.	Comparison between dithiothreitol and ascorbic acid assays, analytical validation of OP online measurement methodology.
Source apportionment	Technique used to identify and quantify the contributions of various pollution sources both at the origin and at a receptor site.	Source apportionment study using Positive Matrix Factor (PMF), Chemical Mass Balance (CMB), or other methods.
Spatial distribution	Use of Geographic Information Systems (GIS) to delimit areas of homogeneous concentration with respect to relevant limit/threshold/alert values.	Use of different statistical analysis techniques (clustering, PCA, correlation) on data obtained from samples spatially distributed within a given area.
Modeling air pollution	Selection of monitoring stations representative of geographical areas related to the spatial model resolution; data assimilation; prediction; generation of predictive models from experimental data.	Develop a predictive model for OP levels associated with air pollution in a given area.
Management tool	Development and/or evaluation (economic, compliance, perception) of the management tool.	Replacement of wood-burning stoves, creation of environmental corridors for public transportation, and other management tool.
Other	Other objectives or strategies that has not yet been a considered and has a low percentage of occurrence.	Objectives studies not included in previous examples.

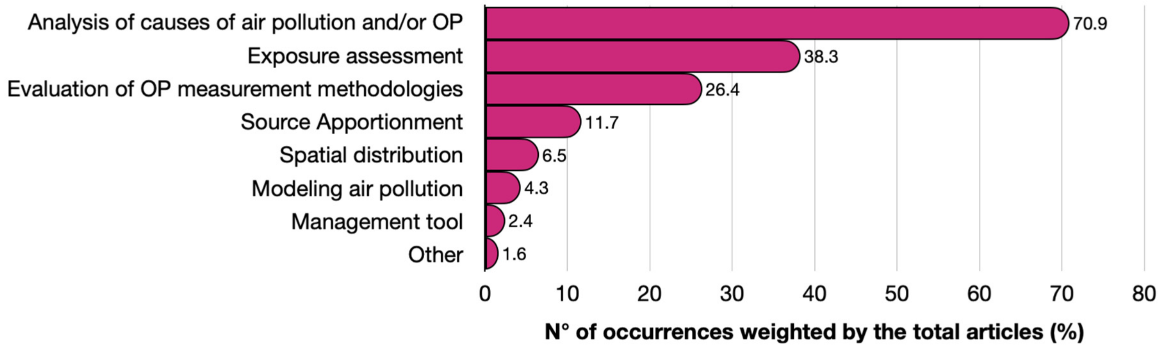

Figure S4. Histogram of occurrences related to objectives of the study, weighted by total collected articles (see Table S4 for additional information).

Table S5. The normalized keywords used related to type of OP assay from article collection, brief descriptions, and examples.

Type of OP assay	Brief descriptions	Examples
Acellular	Evaluation of the OP of PM without the use of live cells by monitoring chemical reactions in vitro.	Measurement of the OP of PM using the DTT Assay.
Cellular	Evaluation of the OP of PM by analyzing the responses of cells exposed to PM.	Measurement of the OP of PM using DCFH-DA probe or detection of oxidative stress markers in cells.

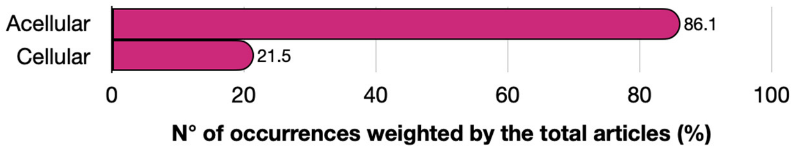

Figure S5. Histogram of occurrences related to type of OP assay, weighted by total collected articles (see Table S5 for additional information).

Table S6. The normalized keywords used related to OP assay from article collection, target molecule or method name, measurement principle, and reference.

OP assay	Target molecule (common name) or method	Measurement principle	Reference
DTT	1,4- Dithiothreitol.	Measurement of the oxidation/consumption rate of dithiothreitol.	[1]
AA	Ascorbic acid.	Measurement of oxidation/consumption rate of ascorbate.	[1]
DCFH	2',7'-Dichlorofluorescein.	Measurement of fluorescence produced by the conversion of 2,7-dichlorofluorescein diacetate (DCFH-DA) to the fluorescent compound 2,7-dichlorofluorescein (DCF) upon exposure to reactive oxygen species (ROS).	[1]
GSH	Reduced glutathione.	measurement of the oxidation/consumption rate of reduced glutathione.	[1]
RTLF	Respiratory Tract Lining Fluid assay.	Measurement of depletion of specific antioxidants in a synthetic respiratory tract lining fluid (RTLF) model.	[1]
EPR/ESR	Electron Paramagnetic/Spin Resonance.	Measurement of hydroxyl radical formation in the presence of hydrogen peroxide.	[1]
MOS	Detection of markers of oxidative stress.	Use analytical methods to detect/quantify oxidative stress markers at the cellular level, such as nitroxide probe in DMSO, cytochrome C or Nrf2/antioxidant response.	[2–4]
BPEAnit	9,10-bis (phenyl ethynyl) anthracene-nitroxide.	Detection of free radicals such as peroxyl and hydroxyl.	[5]
UA	Uric acid.	Measurement of depletion of Uric Acid.	[1]
PSA	Plasmid scission assay.	Use of plasmid to detect the formation of DNA strand breaks by ROS.	[6]
CRAT	Chemiluminescent Reductive Acridinium Triggering assay.	Use of Chemiluminescence of acridinium esters to quantify rates of hydrogen peroxide production.	[1]
LML	Luminol.	Use of Chemiluminescence of Luminol to characterization of OP.	[7]
DHE	Dihydroethidium.	Specific assay to detect superoxide radical, separating of the products by HPLC.	[8]
Other	Other assays that have not yet been a considered and has a low percentage of occurrence.	Objectives studies not included in previous examples, such as Cytochrome- C reduction rate.	

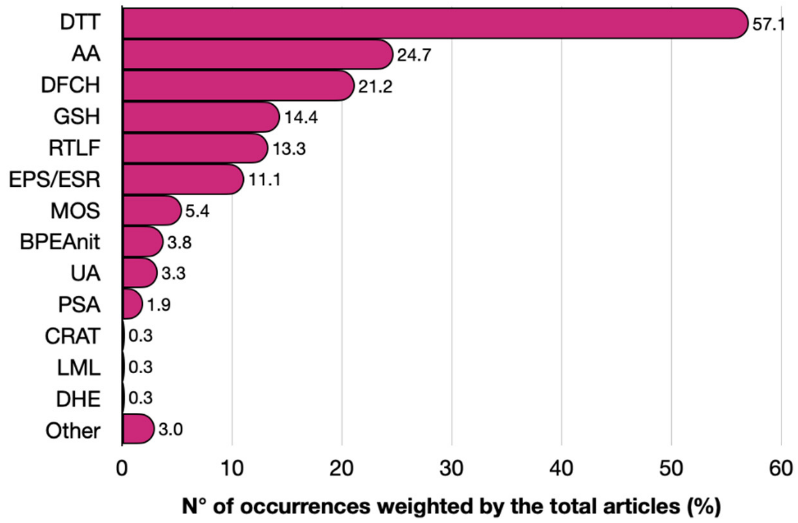

Figure S6. Histogram of occurrences related to OP assay, weighted by total collected articles (see Table S6 for additional information).

Table S7. The normalized keywords used related to evaluation method complementary to the OP, brief descriptions, and examples.

**Evaluation method complementary to the OP**	**Brief description**	**Examples**
Concentrations	Measurement of the abundance of a particular pollutant in the air.	concentration of any species or chemical compound.
Chemical composition	Determination of the chemical composition of PM.	Elemental composition of PM, Elemental/organic carbon content in PM (see Table S8).
Toxicological	Study of direct health effects of air pollutants to characterize their toxicity (i.e., determine mechanisms of action, evaluate thresholds, acute and chronic effects of pollutants).	Dose-response studies in cell culture or model organisms exposed to air pollutants.
Meteorological measurements	Meteorological parameters measurement.	Temperature, relative humidity, atmospheric pressure, wind speed/direction.
Physical measurements of pollutants	Measurement or characterization of physical properties of contaminants.	Size distribution or morphology of PM.
Computer Modeling	Use of mathematical-computational models to simulate and predict the dynamics of factors that regulate air quality.	HYSPLIT model for air mass trajectories, Land use regression models, and other mathematical-computational models.
Epidemiological	Evaluation of the potential effects of air pollution on the incidence of disease or other health effects of the exposed population.	Relationship between increased concentration of respirable PM and the incidence of cancer or other disease.
Biological composition	Characterization of biological elements present in PM.	Pollen, virus, bacteria, fungi, or endotoxin.
Satellite approach	Use of satellite data	MODIS-MAIAC (AOD) or other satellite images.
Other	Other evaluation methods that have not yet been a considered and has a low percentage of occurrence.	Evaluation methods not included in previous examples.

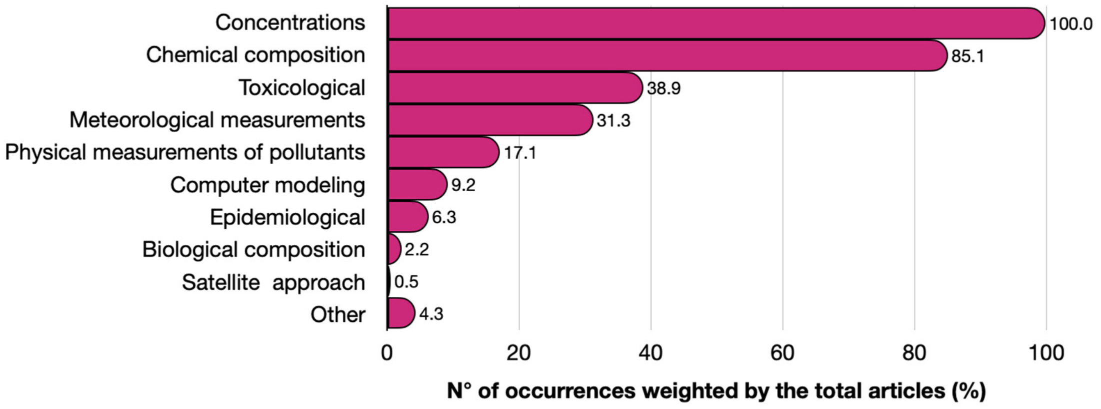

Figure S7. Histogram of occurrences related to evaluation method complementary to the OP, weighted by total collected articles (see Table S7 for additional information).

Table S8. The normalized keywords used related to contaminant or variable other than OP, brief descriptions, and examples.

**Contaminant or variable other than OP**	**Brief descriptions**	**Examples**
Particulate matter	Mixture of solid and liquid particles suspended in the air, which, based on their size diameter, are classified as PM_{10}, $PM_{2.5}$ or PM_1 (particles with a diameter of 10, 2.5 and 1 micrometers or less, respectively), among others.	PM_{10}, $PM_{2.5}$, PM_1.
Elemental composition	Characterization of elemental composition (heavy metals, metalloids, etc.) of the PM.	Hg, As, Cu, Ca, Si, and another element.
Carbon compounds	Determination of the content of the different carbonaceous species present in the PM.	Elemental carbon (EC), organic carbon (OC), brown carbon (Brc) or black carbon (BC).
Anions and Cations	Determination of the content of anions and cations in PM.	K^+, Ca^{+2}, Cl^-, NO_2^{-3}
Biological end-point	Biological response to exposure to air pollutants.	Cellular assay, production of proteins and/or compounds in response to exposure to PM.
Polycyclic aromatic hydrocarbons	Chemical compounds with multiple aromatic rings in their structure.	Naphthalene, Fluoranthene, and other Polycyclic aromatic hydrocarbons.
Nitrogen oxides	Reactive gases composed of nitrogen and oxygen atoms, encompasses various compounds. Often expressed as NOx.	Nitrogen monoxide (NO), Nitrogen dioxide (NO_2).
Volatile/semivolatile organic compounds	Organic compounds that have a high vapor pressure at ambient conditions therefore readily evaporate into the atmosphere. Expressed as VOCs/SVOCs in studies.	Methane, Ethylene, Toluene, and other VOCs/SVOCs.
Ozone	It refers to the determination of the ozone concentration at the tropospheric level.	Evaluation of the vertical profile of the ozone concentration.
Quinones	Aromatic compounds with two carbonyl groups in adjacent positions on the aromatic ring.	Naphthoquinones, Anthraquinones, Benzoquinone, and other quinones.
Carbon oxides	Chemical compounds composed of carbon and oxygen atoms. Often expressed as COx.	COx, CO, CO_2.
Secondary Organic Aerosols	Fraction of atmospheric aerosols formed by oxidation and condensation of volatile organic compounds on pre-existing particles.	based on their source: Anthropogenic SOA, Biogenic SOA.
Sulfur oxides	Chemical compounds composed of sulfur and oxygen atoms. Often expressed as SOx.	Sulfur dioxide (SO_2), sulfur trioxide (SO3).
Biological	Characterization of biological elements present in the PM.	Pollen, viruses, bacteria, fungi and endotoxin.
Emergent contaminants	Group of pollutants that are not commonly monitored in the environment because they have been recently recognized or	Microplastics, nanomaterials, Pharmaceutical products.

	detected and are of increasing concern due to their possible adverse effects on human health and the environment.	
Other	Other contaminant or variable that has not yet been a consideration and has a low percentage of occurrence	Specific organic compounds not considered in previous categories.
No information	Not reported in the article.	Not reported in the article.

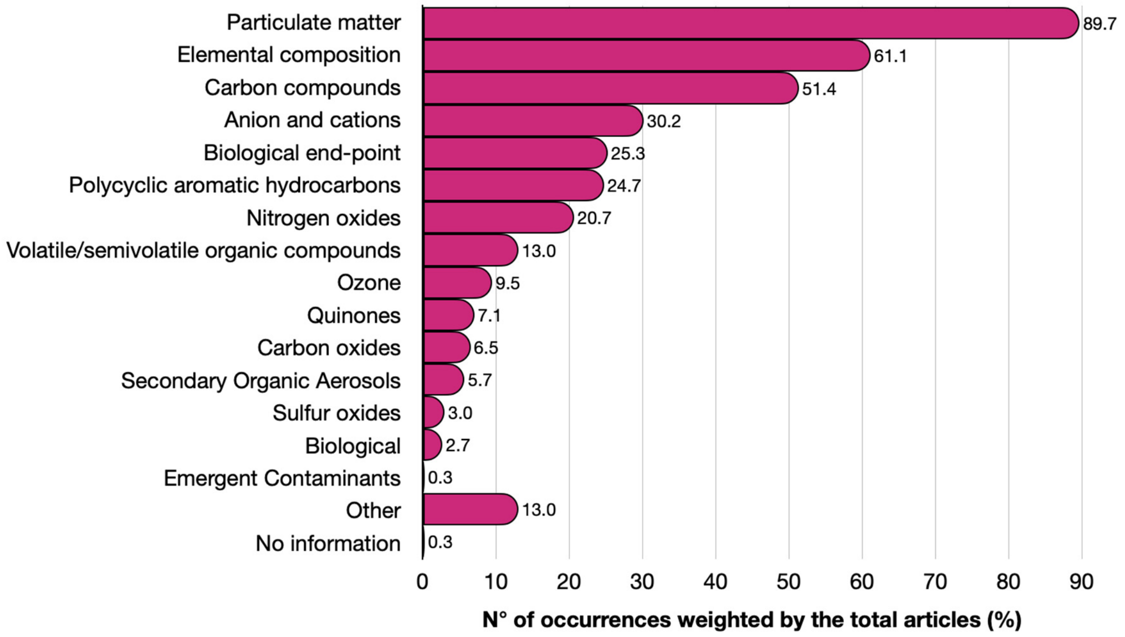

Figure S8. Histogram of occurrences related to contaminant or variable other than OP, weighted by total collected articles (see Table S8 for additional information).

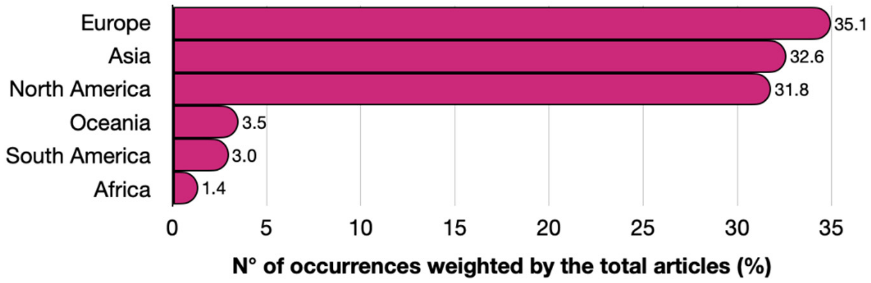

Figure S9. Histogram of occurrences related to continent of PM samples, weighted by total collected articles.

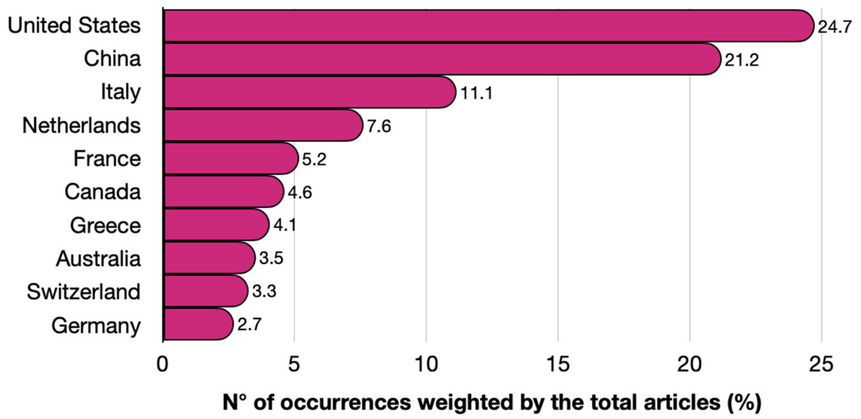

Figure S10. Histogram of occurrences related to country of PM samples, weighted by total collected articles.

Table S9. The normalized keywords used related to emission source from article collection, brief descriptions, and examples.

Emission source	Brief descriptions	Examples
Traffic	Source of mobile emissions from vehicle engine combustion.	Public transport, vehicles, motorcycles, or other motor vehicles.
Domestic heating/wood burning	Point source that includes heating activities.	Heating with stoves or fireplaces, woodburning or gas burning.
Industrial and commercial emissions	Point source that includes industrial or commercial activities.	Smelters, food production, and other industries.
unspecified biomass burning	Emission source from biomass combustion not specifically identified or categorized in the study.	Studies that refer to the burning of biomass without specifying its origin.
Natural sources	Emission source of natural origin.	Soil resuspension by wind action, volcanic eruption, marine aerosols, etc.
Non-exhaust emissions (traffic related)	Mobile source associated with vehicular traffic, other than exhaust emissions.	Brake or tire wear.
Power generation	Point source emissions from power/electricity generation.	Thermoelectric, hydroelectric, and other combustion-based power generation sources.
Agriculture	Emission sources from agricultural activities.	Grassland burning or use of pesticides.
Wildfires	Emission source produced by forest fires.	Forest fires that occur during warm and drought conditions.
Mining	Emission sources from mining activities.	Suspended dust from mining and transporting minerals, tailings, and Smelters.
Other	Other emission sources that has not yet been a consideration and has a low percentage of occurrence	Emissions from construction activities, fireworks, and other emission sources not listed above.
No information	Not reported in the article.	Not reported in the article.

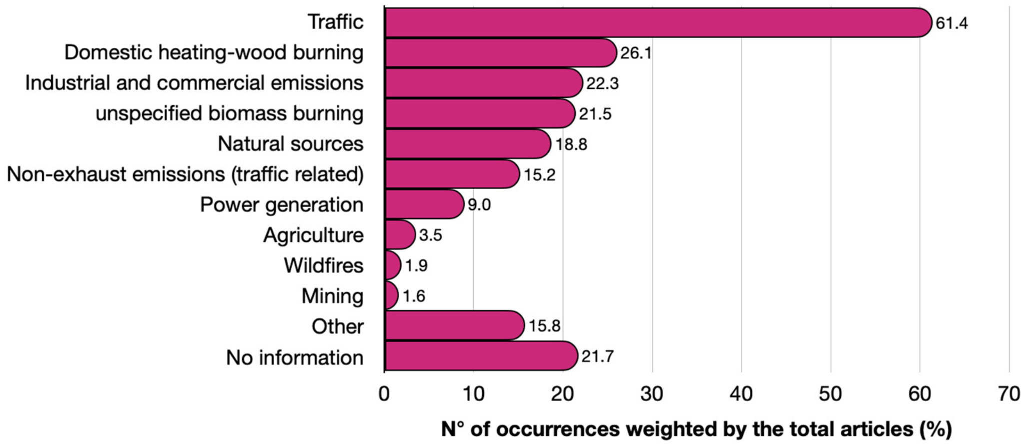

Figure S11. Histogram of occurrences related to emission source, weighted by total collected articles (see Table S9 for additional information).

Table S10. The normalized keywords used related to temporal scales of the study, brief descriptions, and examples.

Temporal scales	Brief descriptions	Examples
More than a year - 1 year	More than 12 months of sampling or data collection.	Evaluation of trends or changes from year to year.
Year - Season	More than 4 to 12 months of sampling or data collection.	Assessing seasonal effects.
3 months - 1 month	Less than 4 months, usually between 1 and 3 months, sampling or collecting data that extends beyond 4 months.	Monthly, weekly and day-to-day variability.
3 weeks - Hours	Short-term, usually less than 1 month, sampling or data collection campaigns.	Assessment of events on a short time scale (fires, fireworks, gas leaks, explosions, or events of comparable duration).
Other	Other time scales that has not yet been a consideration and has a low percentage of occurrence	Monitoring rapid changes in laboratory conditions (e.g. temperature or pH fluctuations) or capturing events that happen in split seconds (reaction mechanism).
No information	No report in the article.	No report in the article.

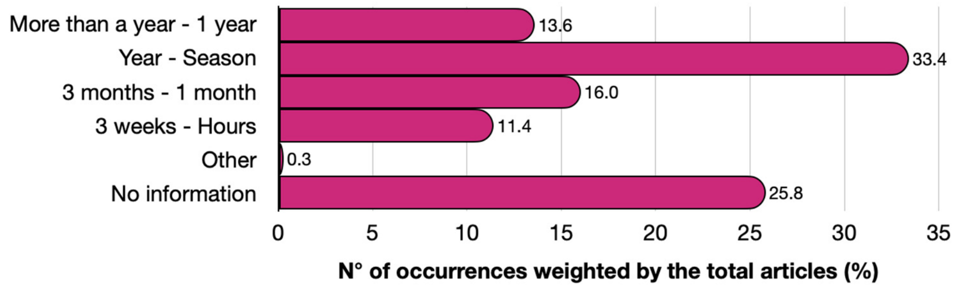

Figure S12. Histogram of occurrences related to temporal scales, weighted by total collected articles (see Table S10 for additional information).

Table S11. The normalized keywords used related to spatial scales of the study, brief descriptions, and examples.

Spatial scales	Brief descriptions	Examples
Global	Studies extending over a range greater than 40,000 km^2.	Studies on a global scale, which may involve comparison between 2 or more countries in different continents.
Regional	Studies extending over a range of 1000 km^2 - 40,000 km^2.	Study of larger scale extension to the local scale, which may involve transport phenomena or spatial distribution between adjacent regions or countries.
Local	Studies extending over a range of 1 km^2 - 100 km^2.	Studies conducted at rural, suburban and urban levels.
Micro-scale	Studies extending over a range of 1 m^2 - 1 km^2.	Studies conducted at indoor level, workplace, residential, street canyons or critical point.
No information	No report in the article.	No report in the article.

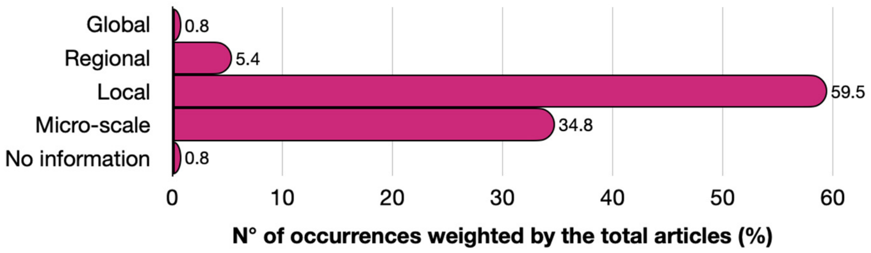

Figure S13. Histogram of occurrences related to spatial scales of the study, weighted by total collected articles (see Table S11 for additional information).

Table S12. The homogenized keywords used to extract potential impact from the study from article collection, brief descriptions, and examples.

**Potential impact of the study**	**Brief descriptions**	**Examples**
Human health	Studies whose results provide scientific evidence explicitly applicable or related to human health.	Epidemiological studies related to air quality and OP; toxicological studies of the effect of exposure to atmospheric pollutants and OP, and other similar studies.
Regulatory and Legal	Studies whose results provide scientific evidence explicitly applicable or related to regulations and legislation (public policies).	Regulatory assessment studies, establishment of baseline contaminant levels and OPs for the creation or modification of quality standards.
Understanding OP mechanism	Studies whose results provide scientific evidence explicitly applicable or related to the understanding of the mechanism underlying OP.	Evaluation of OP response to different reaction conditions, such as type of filter used, extraction methods, storage duration of samples or PM chemical composition.
Technological development	Studies whose results are explicitly applicable to the development of technology focused on improving air quality.	Design of measuring devices (e.g. sensors) and OP online measuring instruments.
Ecological	Studies whose results provide scientific evidence explicitly applicable to or related to protecting ecosystems and biodiversity.	Exposure studies with vegetal or animal species, use of bioindicators for air quality assessment.
Climate	Studies whose results provide scientific evidence explicitly applicable or related to climate change.	Studies of the effects of air pollution on climate or vice versa (climate-air quality relations, modeling or forecasting).
.Other	Other potential impact that has not yet been a consideration and has a low percentage of occurrence	Studies or impacts not covered in previous examples

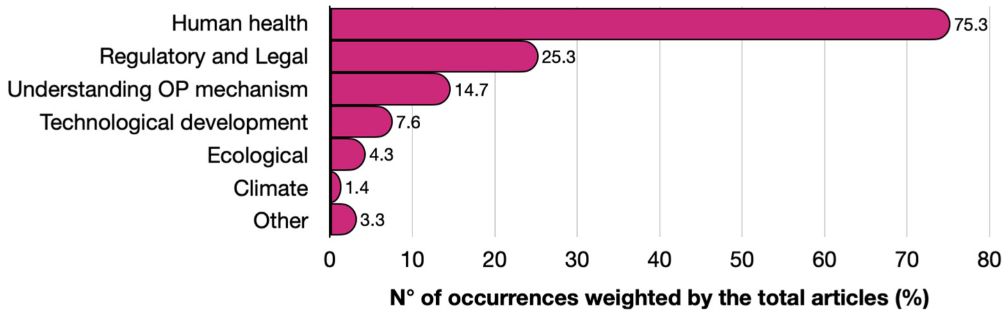

Figure S14. Histogram of occurrences related to potential impacts from the study, weighted by total collected articles (see Table S12 for additional information).

Reference

1. Rao, L.; Zhang, L.; Wang, X.; Xie, T.; Zhou, S.; Lu, S.; Liu, X.; Lu, H.; Xiao, K.; Wang, W.; et al. Oxidative Potential Induced by Ambient Particulate Matters with Acellular Assays: A Review. *Processes* **2020**, *8*, 1410, doi:10.3390/pr8111410.
2. Ciriello, F.; Gualtieri, M.; Longhin, E.; Ruffo, R.; Camatini, M.; Parenti, P. A New Method and Tool for Detection and Quantification of PM Oxidative Potential. *Environ. Sci. Pollut. Res.* **2015**, *22*, 12469–12478, doi:10.1007/s11356-015-4551-2.
3. Park, B.; London, N.R.; Tharakan, A.; Rengasamy, P.; Rajagopalan, S.; Biswal, S.; Pinto, J.M.; Ramanathan, M. Particulate Matter Air Pollution Exposure Disrupts the Nrf2 Pathway in Sinonasal Epithelium via Epigenetic Alterations in a Murine Model. *Int. Forum Allergy Rhinol.* **2022**, *12*, 1424–1427, doi:10.1002/alr.23010.
4. Stevanovic, S.; Miljevic, B.; Eaglesham, G.K.; Bottle, S.E.; Ristovski, Z.D.; Fairfull-Smith, K.E. The Use of a Nitroxide Probe in DMSO to Capture Free Radicals in Particulate Pollution. *European J. Org. Chem.* **2012**, *2012*, 5908–5912, doi:10.1002/ejoc.201200903.
5. Jovanovic, M. V.; Savic, J.Z.; Salimi, F.; Stevanovic, S.; Brown, R.A.; Jovasevic-Stojanovic, M.; Manojlovic, D.; Bartonova, A.; Bottle, S.; Ristovski, Z.D. Measurements of Oxidative Potential of Particulate Matter at Belgrade Tunnel; Comparison of BPEAnit, DTT and DCFH Assays. *Int. J. Environ. Res. Public Health* **2019**, *16*, 4906, doi:10.3390/ijerph16244906.
6. Crobeddu, B.; Aragao-Santiago, L.; Bui, L.-C.; Boland, S.; Baeza Squiban, A. Oxidative Potential of Particulate Matter 2.5 as Predictive Indicator of Cellular Stress. *Environ. Pollut.* **2017**, *230*, 125–133, doi:10.1016/j.envpol.2017.06.051.
7. Morozzi, P.; Bolelli, L.; Brattich, E.; Ferri, E.N.; Girotti, S.; Sangiorgi, S.; Orza, J.A.G.; Piñero-García, F.; Tositti, L. Chemiluminescent Fingerprints from Airborne Particulate Matter: A Luminol-Based Assay for the Characterization of Oxidative Potential with Kinetical Implications. *Sci. Total Environ.* **2021**, *789*, 148005, doi:10.1016/j.scitotenv.2021.148005.
8. Forman, H.J.; Finch, C.E. A Critical Review of Assays for Hazardous Components of Air Pollution. *Free Radic. Biol. Med.* **2018**, *117*, 202–217, doi:10.1016/j.freeradbiomed.2018.01.030.

Disclaimer/Publisher's Note: The statements, opinions and data contained in all publications are solely those of the individual author(s) and contributor(s) and not of MDPI and/or the editor(s). MDPI and/or the editor(s) disclaim responsibility for any injury to people or property resulting from any ideas, methods, instructions or products referred to in the content.
