# Supplementary material for: The Oxidative Potential of Airborne Particulate Matter Research Trends, Challenges, and Future Perspectives—Insights from a Bibliometric Analysis and Scoping Review
Source: Antioxidants (Basel). 2024 May 24;13(6):640. doi: 10.3390/antiox13060640 (PMC11200927; doi:10.3390/antiox13060640)
Supplement: Supplementary file 1 [file antioxidants-13-00640-s001.zip › BibOPPM_Protocol-ScR.pdf]

antioxidants MDPI

Supplementary Materials – Protocol scoping review according to PRISMA-ScR Checklist

Oxidative Potential of Airborne Particulate Matter Research Trends, Challenges, and Future Perspectives - Insights from a Bibliometric Analysis and Scoping Review. Protocols

Luis Felipe Sánchez, Loreto Villacura, Francisco Catalán, Richard Toro A., Manuel A. Leiva G. *

Departamento de Química, Facultad de Ciencias, Universidad de Chile, Las Palmeras 3425, Ñuñoa, Santiago, Chile.

* Correspondence: manleiva@uchile.cl

Abstract

Background: Scientific research has established a direct association between particulate matter (PM) exposure and adverse health effects. In addition, it has been documented that PM can induce oxidative stress at the cellular level. The oxidative potential (OP) arises as a metric of this capacity and represents a novel approach that allows a comprehensive assessment of adverse health effects induced by PM.

Objectives: Through a bibliometric analysis and scoping review of the existing literature related with the Oxidative Potential of Particulate Matter (OP-PM), this study aims to clarify the current state of the field and propose possible directions for future research.

Methods: The database used is the Web of Science (WoS) Core Collection. The search strategy involves a series of previously designed search queries. After duplicate verification, we only included reports that measured OP in PM samples or evaluated and developed methods for measuring OP, based on the established eligibility criteria. We utilized a questionnaire to obtain detailed information on OP-PM in the selected studies. During the data charting process, a collaborative web-application was used to record the progress of each reviewer and facilitate panel discussions.

Results: Of the 569 articles screened, 368 were selected for further analysis. OP is an emerging field of study with a remarkable increase in the number of publications over the last decade. These are mainly concentrated in eight journals and a few academic institutions. Key research hotspots for OP-PM include capacity building, interdisciplinary collaboration, understanding emission sources and atmospheric processes, and the impacts of PM and its OP.

Conclusions: Highlights the growing importance of OP-PM research and the need for increased collaboration and interdisciplinary approaches. Key contributors, institutions, and journals are identified, providing valuable insights into research hotspots and future directions. The study enhances the understanding of the risks associated with PM and supports the development of targeted mitigation strategies to protect public health and environmental sustainability in a climate change scenario.

Keywords: airborne; particulate matter; oxidative potential; oxidative stress; paradigm.

Introduction

During the past few decades, a growing body of epidemiologic evidence has established an incontrovertible link between exposure to particulate matter (PM) and adverse health outcomes [1]. These health effects are associated not only with the mass concentration of PM, but also with its chemical composition.[2]. Due to its complexity, the underlying

mechanism describing the toxicity of PM remains unclear [3,4]. However, a recognized hypothesis is that PM generates reactive species that produce oxidative stress at the cellular level, triggering an inflammatory response [5,6]. Within this conceptual framework, the notion of oxidative potential (OP), or the redox activity of PM, emerges as a descriptor and metric of the relationship between the chemical composition of PM, its physical properties, and the possible toxicological effects it may exert on human health upon exposure [7]. Thus, OP is closely related to the biological mechanisms by which adverse health effects of PM constituents can manifest themselves [8].

Considerable progress has been made in the measurement of OP in recent years. This progress is an important step toward establishing OP as an innovative and promising measure. Remarkable progress has been made in the design and standardization of toxicity assays, as methodologies have been refined [9]. Significant advances are being made in understanding how various components of particulate matter can contribute to their oxidative potential and hence their ability to cause oxidative damage in human tissues [10]. These discoveries, provide a solid framework for future research in this rapidly evolving field. Finally, we must emphasize that this progress also allows the development of effective strategies to mitigate the harmful effects of air pollution on the health of the population.

Aim

The main objective of the present research is to identify the main points of interest in the study of Oxidative Potential of Particulate Matter and its environmental and health impacts (OP-PM). Through a bibliometric analysis and scoping review of the existing literature, this study aims to clarify the current state of the field and propose possible directions for future research.

Methods and analysis.

To answer our main objective, we will conduct a careful bibliometric analysis and scoping review of current knowledge. As demonstrated in the academic literature, such bibliometric studies facilitate a clear delineation of scientific research trajectories and meticulously examine the current state of a given discipline or research area [11]. Additionally, we utilized the 2018 extension of the PRISMA framework, specifically tailored for scoping reviews. This framework comprises a series of well-established steps, incorporating clear inclusion and exclusion criteria, along with a rigorous quality assessment [12]. The steps we followed encompass: i) defining the field, ii) developing the search strategy and selecting databases, iii) refining the search and eligibility criteria, iv) conducting quantitative and qualitative analysis, and v) visualizing the results.

Identifying the research question

Significant advancements have been made in recent years regarding the measurement of OP, a metric that is emerging as a novel approach that allows for a comprehensive assessment of adverse health effects induced by PM. These advances provide a solid framework for future research in this rapidly evolving field. Based on this background, previous literature reviews, and our experience in conducting experimental research in this area, we formulated a broad research question: What are the trends, challenges, and future perspectives in the field of Oxidative Potential of Airborne Particulate Matter Research?

Eligibility criteria

Only reports that measured OP in PM samples or evaluated and developed methods for measuring OP and were published by December 31, 2021 were included. Reports that consisted of reviews, meta-analyses, bibliometrics analyses or similar were excluded, as were reports were not within the scope of the search.

Types of study

Only studies classified as "Article" in Web of Science (WoS) are considered.

Databases

The database selected in the present study is the Web of Science (WoS) Core Collection. This database, which is often used in such studies, integrates several databases, including the Science Citation Index Expanded (SCI-Expanded), the Social Sciences Citation Index (SSCI), the Arts & Humanities Citation Index (AHCI), and the Emerging Sources Citation Index (ESCI).

Search strategy

The search strategy within the WoS was implemented through a series of seven meticulous search queries, each using unique groups of keywords that were agreed upon by consensus by all authors (see Table 1).

Table 1. Keyword sets and search strategy used in the study.

	**Keyword Set #1**
	("pollut*" AND air) OR (atmosp* AND pollut*) OR ("bad air quality"); ("Ambient air pollution") OR ("quali*" NEAR air)
	**Keyword Set #2**
	("partic* matter" OR "fine particulate" OR particulate OR "ultrafine partic*" OR "ultrafine partic*" OR "partic* pollut*" OR aeroso* OR Size segreg* OR Ozone OR Smog OR ("photochemical" NEAR smog) OR "nitrogen *oxide*" OR "sulfur *oxide*" OR "carbon monoxide" OR "heavy metal*" OR "volatile organic gases" OR "organic gases" OR dioxi* OR fura* OR "polycyclic aromatic hydrocarbon*" OR "Polychlorinated biphenyl*" OR "persistent organic pollutant*" OR "volatile organic compound*" OR "quinone*" OR AA*depletion OR GSH*depletion OR “Congo Red” OR “DCF” OR “DCFH-DA” OR “2-deoxyribose” OR DHE OR DTT OR ESR OR EPR OR Luminol OR CRAT OR “2′,7′-dichlorofluorescin” OR “dithiothreitol” OR “acid ascorbic” OR “Dihydroethidium” OR “glutathione” OR “electron spin resonance” OR “electron paramagnetic resonance”)
	**Keyword Set #3**
	**(oxidative NEAR/5 potential)**
**Search N°**	**Search query**
1	TITLE, ABSTRACT AND AUTHOR KEYWORDS: TI= (Keyword Set #1) OR AB= (Keyword Set #1) OR AK= (Keyword Set #1) with the options Search in: Web of Science Core Collection; Edition: All; Publication date: All years (1975-present).
2	TITLE, ABSTRACT AND AUTHOR KEYWORDS: TI= (Keyword Set #2) OR AB= (Keyword Set #2) OR AK= (Keyword Set #2) with the options Search in: Web of Science Core Collection; Edition: All; Publication date: All years (1975-present).
3	TITLE, ABSTRACT AND AUTHOR KEYWORDS: TI= (Keyword Set #3) OR AB= (Keyword Set #3) OR AK= (Keyword Set #3) with the options Search in: Web of Science Core Collection; Edition: All; Publication date: All years (1975-present).
4	#1 OR #2 (Merge Search N°1 and Search N°2)
5	#3 AND #4 (Combine Search N°3 and Search N°4)
6	[Refine] by Document Types: Article
**7**	[Exclude] by Publication Years: 2022-present

Searches #1, #2, and #3 correspond to independent queries that, for each set of keywords separately, include the fields tags title (TI), abstract (AB), and author keywords (AK), linked by the Boolean operator 'OR'. These searches are performed across all available issues and publication years (1975-present). In search #4, searches #1 and #2 are merged

by using the Boolean operator “OR”. Next, in query #5, queries #4 and #5 are combined using the “AND” Boolean. Finally, in queries #6 and #7, the results are filtered by adding "articles" only and excluding articles published after 2022, respectively. At the end of the process, a database report was obtained.

Study selection

Duplicate verification using an automated tool and evaluation by three reviewers (LFS, FC, and LV) was used to refine the report database. These reviewers independently determine the eligibility of a report by examining the title, abstract, keywords of the author, and/or the full report. To be considered eligible for a report, the three reviewers must agree that the report is eligible based on the eligibility criteria. In case of disagreement, the report is given a "conflict" status and must be reviewed by a fourth reviewer (MALG) and discussed in a panel. The final decision on eligibility is made by a majority of reviewers. These process was facilitated by a Web-enabled collaborative review application, SWIFT ActiveScreener, provided by SCIME [13]. This application includes a deduplication function that verifies the presence of duplicates when uploading the report database based on the title, journal and/or year of publication.

Data extraction

To conduct the bibliometric analysis, we completed missing fields (such as DOI, Author keywords, Journals, etc) by extracting information from available articles when bibliographic data was incomplete or unclear. This control measure enhances the level of certainty and confidence in the information in the database.

During the review process, a questionnaire was used to obtain detailed information on OP-PM in the selected reports, with which homogenized keywords were assigned to each report (details of these keywords are provided in the Supplementary Material, Table S1). These keywords consider aspects such as author affiliation, study characteristics, study objectives, type of OP assay, complementary assessment method to OP, pollutants or variables other than OP, spatiotemporal scales, spatial scale, emission source contributing to OP, and potential implications of the study. To minimize the risk of bias and increase confidence in the results, homogenized keywords were discussed and agreed in a panel by all reviewers. Categorical variables were designed to represent a range of values and extract numerical data, such as spatiotemporal scales. Furthermore, an “other” option was included next to a text box in each question of the questionnaire to allow comments regarding information not initially considered. All reviewers on the panel reviewed these comments. If the level of occurrence warranted it, new homogenized keywords were assigned and the corresponding correction was applied to the reports reviewed previously. Comments with weighted occurrences of less than 1% were classified under the "other" option. This method enables the acquisition of more precise information, thus minimizing potential heterogeneity in the results of individual reports.

Data synthesis

VOSviewer version 1.6.19 [14] and the R-based web interface Bibliometrix version 3.2.1 [15] were used to construct and visualize bibliometric networks for quantitative and qualitative bibliometric analyses. Within the R environment [16], the Ggplot 2 package R [17] was used to generate specific plots. This comprehensive approach integrates different tools and methods to elucidate patterns and networks and provides insightful representations of bibliographic data.

Within the bibliometric analysis, a bibliometric summary was created for the collection of articles along with a time series of articles published on the OP-PM (see Figure 1b, c). Figure 2 include: a) a Histogram and cartogram of geographical distribution of articles according to country of authors and coauthors, b) histogram and cartogram of geographical distribution of articles according to country of corresponding author(s) and

c) origin-destination diagram showing collaboration between corresponding authors from one country with respect to others. Figure 3 include: a) Bradford's law of most prominent sources. b) Number of published articles (NP) per journal. c) Total number of local citations (TC) per journal. d) Impact factor (IF) of the journal. e) H-index of journal considering the articles of the collection on the OP-PM. Figure 4 include a) Frequency distribution of publications using Lotka's law. b) Ranking of the most relevant authors according to the number of published articles (NP), c) Number of total local citations (LC) and d) H index. e) The cooperation networks of the productions of the authors. Table 2 shows the top 10 relevant publications in research on OP-PM based on the number of local citations (LC). In each case, the number of global citations (GC) and the percentage of the LC/GC ratio are indicated. And finally, Figure 6 presents an analysis of hotspots in OP del PM from 2003 to 2021. This analysis is based on: a) Cooccurrence network of WoS subject categories, and b) Cooccurrence network of author keywords.
The findings resulting from the questionnaire applied to each of the selected articles are described in Section 3.7. "Research hotspot analysis," in the manuscript. Figures supporting this analysis and a brief description of the categories used are provided in the supplementary material. Table S1: Set of homogenized keywords assigned to each article according to the specific characteristics of the study through panel review. Tables S2 – S12: Homogenized keywords used to extract information from the study from article collection, brief descriptions, and examples. Figures S2 – S14: Histogram of occurrences related to homogenized keywords, weighted by total collected articles.

Conclusion

Overall, this bibliometrics analysis and scoping review highlights the growing importance of OP-PM research and the need for increased collaboration and interdisciplinary approaches. It identifies key contributors, institutions, and journals in the field and provides insights into research hotspots and future directions. The study contributes to a more comprehensive understanding of the risks associated with PM and supports the development of targeted mitigation strategies to protect public health and environmental sustainability in a climate change scenario.

Ethics approval and consent to participate. This study did not require ethics approval or consent to participate, as it exclusively utilized available data obtained from established databases. No experiments on humans, animals, or in vivo were conducted as part of this research.

Author Contributions: L.F.S.P: Conceptualization, Data Curation, Formal analysis, Visualization**,** Methodology, Investigation, Writing, **F.C.:** Data Curation, Formal analysis, Visualization, **L.V.:** Data Curation, Formal analysis, Visualization, **R.T.A.:** Conceptualization, Editing, **M.L.G.:** Founding, Conceptualization, Methodology, Investigation, Writing - Reviewing and Editing.

Funding: This research was partial funded by Vicerrectoría de Investigación y Desarrollo (VID), Universidad de Chile, Programa de Apoyo a Proyectos de Enlace con Concurso Fondecyt Regular VID 2020 and 2023, grant nº ENL17/20 and ENL21/23, and Chilean National Fund for Scientific and Technological Development (FONDECYT) ANID FONDECYT Regular Grant No. 1220948; LV acknowledges support from ANID: 2021 National Doctoral degree Scholarship Program, no. Nº 21212276. LFS acknowledges support from ANID, CONICYT: 2019 National Doctoral degree Scholarship Program, no. 21191906.

Acknowledgments: The authors acknowledge and are grateful for the contribution of Sciome (https://www.sciome.com) for providing free of charge the SWIFT-Active Screener software, which allowed a fast and efficient review of the articles included in this study.

Conflicts of Interest: The authors declare no conflict of interest.

References

1. Brook, R.D.; Rajagopalan, S.; Pope, C.A.; Brook, J.R.; Bhatnagar, A.; Diez-Roux, A. V.; Holguin, F.; Hong, Y.; Luepker, R. V.; Mittleman, M.A.; et al. Particulate Matter Air Pollution and Cardiovascular Disease. *Circulation* **2010**, *121*, 2331–2378, doi:10.1161/CIR.0b013e3181dbece1.
2. Raaschou-Nielsen, O.; Beelen, R.; Wang, M.; Hoek, G.; Andersen, Z.J.; Hoffmann, B.; Stafoggia, M.; Samoli, E.; Weinmayr, G.; Dimakopoulou, K.; et al. Particulate Matter Air Pollution Components and Risk for Lung Cancer. *Environ. Int.* **2016**, *87*, 66–73, doi:10.1016/j.envint.2015.11.007.
3. Kelly, F.J.; Fussell, J.C. Size, Source and Chemical Composition as Determinants of Toxicity Attributable to Ambient Particulate Matter. *Atmos. Environ.* **2012**, *60*, 504–526, doi:10.1016/j.atmosenv.2012.06.039.
4. Li, T.; Yu, Y.; Sun, Z.; Duan, J. A Comprehensive Understanding of Ambient Particulate Matter and Its Components on the Adverse Health Effects Based from Epidemiological and Laboratory Evidence. *Part. Fibre Toxicol.* **2022**, *19*, *67*, doi:10.1186/s12989-022-00507-5.
5. Tuet, W.Y.; Fok, S.; Verma, V.; Tagle Rodriguez, M.S.; Grosberg, A.; Champion, J.A.; Ng, N.L. Dose-Dependent Intracellular Reactive Oxygen and Nitrogen Species (ROS/RNS) Production from Particulate Matter Exposure: Comparison to Oxidative Potential and Chemical Composition. *Atmos. Environ.* **2016**, *144*, 335–344, doi:10.1016/j.atmosenv.2016.09.005.
6. Molina, C.; Toro A., R.; Manzano, C.; Canepari, S.; Massimi, L.; Leiva-Guzmán, M. Airborne Aerosols and Human Health: Leapfrogging from Mass Concentration to Oxidative Potential. *Atmosphere (Basel).* **2020**, *11*, 917, doi:10.3390/atmos11090917.
7. Molina, C.; Manzano, C.A.; Toro A., R.; Leiva G, M.A. The Oxidative Potential of Airborne Particulate Matter in Two Urban Areas of Chile: More than Meets the Eye. *Environ. Int.* **2023**, *173*, 107866, doi:10.1016/j.envint.2023.107866.
8. Yang, A.; Jedynska, A.; Hellack, B.; Kooter, I.; Hoek, G.; Brunekreef, B.; Kuhlbusch, T.A.J.; Cassee, F.R.; Janssen, N.A.H. Measurement of the Oxidative Potential of PM2.5 and Its Constituents: The Effect of Extraction Solvent and Filter Type. *Atmos. Environ.* **2014**, *83*, 35–42, doi:10.1016/j.atmosenv.2013.10.049.
9. Charrier, J.G.; Anastasio, C. On Dithiothreitol (DTT) as a Measure of Oxidative Potential for Ambient Particles: Evidence for the Importance of Soluble Transition Metals. *Atmos. Chem. Phys.* **2012**, *12*, 9321–9333, doi:10.5194/acp-12-9321-2012.
10. Gao, D.; Ripley, S.; Weichenthal, S.; Godri Pollitt, K.J. Ambient Particulate Matter Oxidative Potential: Chemical Determinants, Associated Health Effects, and Strategies for Risk Management. *Free Radic. Biol. Med.* **2020**, *151*, 7–25, doi:10.1016/j.freeradbiomed.2020.04.028.
11. José de Oliveira, O.; Francisco da Silva, F.; Juliani, F.; César Ferreira Motta Barbosa, L.; Vieira Nunhes, T. Bibliometric Method for Mapping the State-of-the-Art and Identifying Research Gaps and Trends in Literature: An Essential Instrument to Support the Development of Scientific Projects. In *Scientometrics Recent Advances*; Kunosic, S., Zerem, E., Eds.; IntechOpen: London, 2019 ISBN 978-1-78984-712-3.
12. Tricco, A.C.; Lillie, E.; Zarin, W.; O'Brien, K.K.; Colquhoun, H.; Levac, D.; Moher, D.; Peters, M.D.J.; Horsley, T.; Weeks, L.; et al. PRISMA Extension for Scoping Reviews (PRISMA-ScR): Checklist and Explanation. *Ann. Intern. Med.* **2018**, *169*, 467–473, doi:10.7326/M18-0850.
13. Howard, B.E.; Phillips, J.; Tandon, A.; Maharana, A.; Elmore, R.; Mav, D.; Sedykh, A.; Thayer, K.; Merrick, B.A.; Walker, V.; et al. SWIFT-Active Screener: Accelerated Document Screening through Active Learning and Integrated Recall Estimation. *Environ. Int.* **2020**, *138*, *105623*, doi:10.1016/j.envint.2020.105623.

14. van Eck, N.J.; Waltman, L. Software Survey: VOSviewer, a Computer Program for Bibliometric Mapping. *Scientometrics* **2010**, *84*, 523–538, doi:10.1007/s11192-009-0146-3.
15. Aria, M.; Cuccurullo, C. Bibliometrix : An R-Tool for Comprehensive Science Mapping Analysis. *J. Informetr.* **2017**, *11*, 959–975, doi:10.1016/j.joi.2017.08.007.
16. Posit team. 2022. RStudio: Integrated Development Environment for R (Version 2022.07.1+554). Boston, Massachusetts. R Foundation for Statistical Computing.
17. Wickham, H. *Ggplot2: Elegant Graphics for Data Analysis*; Gentleman, R., Hornik, K., Parmigiani, G., Eds.; 1st ed.; Springer: New York, 2009; ISBN 978-0-387-98140-6.
